# Long noncoding RNA *Smyca* coactivates TGF-β/Smad and Myc pathways to drive tumor progression

**DOI:** 10.1186/s13045-022-01306-3

**Published:** 2022-07-06

**Authors:** Hsin-Yi Chen, Shu-Jou Chan, Xinxin Liu, An-Chi Wei, Ru-In Jian, Kuan-Wei Huang, Yaw-Dong Lang, Jou-Ho Shih, Chun-Chieh Liao, Chiu-Lin Luan, Yu-Tung Kao, Shang-Yin Chiang, Pei-Wen Hsiao, Yuh-Shan Jou, Yunching Chen, Ruey-Hwa Chen

**Affiliations:** 1 Graduate Institute of Cancer Biology and Drug Discovery, College of Medical Science and Technology, Taipei Medical University, Taipei, 110 Taiwan; 2 Ph.D. Program for Cancer Molecular Biology and Drug Discovery, College of Medical Science and Technology, Taipei Medical University, Taipei, 110 Taiwan; 3 Institute of Biological Chemistry, Academia Sinica, Taipei, 115 Taiwan; 4 Institute of Biochemical Sciences, College of Life Science, National Taiwan University, Taipei, 106 Taiwan; 5 Institute of Biomedical Engineering and Frontier Research Center On Fundamental and Applied Sciences of Matters, National Tsing Hua University, Hsinchu, 300 Taiwan; 6 Institute of Biomedical Sciences, Academia Sinica, Taipei, 115 Taiwan; 7 Cancer Progression Research Center, National Yang Ming Chiao Tung University, Taipei, 112 Taiwan; 8 Institute of Molecular Medicine, College of Medicine, National Taiwan University, Taipei, 106 Taiwan; 9 Genome and Systems Biology Degree Program, College of Life Science, National Taiwan University, Taipei, 100 Taiwan; 10 School of Medicine, College of Medicine, Taipei Medical University, Taipei, 110 Taiwan; 11 Agricultural Biotechnology Research Center, Academia Sinica, Taipei, 115 Taiwan

**Keywords:** LncRNA, TGF-β, Smad, c-Myc, EMT, Metastasis, Chemoresistance

## Abstract

**Background:**

Metastasis and chemoresistance are major culprits of cancer mortality, but factors contributing to these processes are incompletely understood.

**Methods:**

Bioinformatics methods were used to identify the relations of *Smyca* expression to clinicopathological features of human cancers. RNA-sequencing analysis was used to reveal *Smyca*-regulated transcriptome. RNA pull-down and RNA immunoprecipitation were used to examine the binding of *Smyca* to Smad3/4 and c-Myc/Max. Chromatin immunoprecipitation and chromatin isolation by RNA purification were used to determine the binding of transcription factors and *Smyca* to various gene loci, respectively. Real-time RT-PCR and luciferase assay were used to examine gene expression levels and promoter activities, respectively. Xenograft mouse models were performed to evaluate the effects of *Smyca* on metastasis and chemoresistance. Nanoparticle-assisted gapmer antisense oligonucleotides delivery was used to target *Smyca* in vivo.

**Results:**

We identify lncRNA *Smyca* for its association with poor prognosis of many cancer types. *Smyca* potentiates metabolic reprogramming, migration, invasion, cancer stemness, metastasis and chemoresistance. Mechanistically, *Smyca* enhances TGF-β/Smad signaling by acting as a scaffold for promoting Smad3/Smad4 association and further serves as a Smad target to amplify/prolong TGF-β signaling. Additionally, *Smyca* potentiates c-Myc-mediated transcription by enhancing the recruitment of c-Myc/Max complex to a set of target promoters and c-Myc binding to TRRAP. Through potentiating TGF-β and c-Myc pathways, *Smyca* synergizes the Warburg effect elicited by both pathways but evades the anti-proliferative effect of TGF-β. Targeting *Smyca* prevents metastasis and overcomes chemoresistance.

**Conclusions:**

This study uncovers a lncRNA that coordinates tumor-relevant pathways to orchestra a pro-tumor program and establishes the clinical values of *Smyca* in cancer prognosis and therapy.

**Supplementary Information:**

The online version contains supplementary material available at 10.1186/s13045-022-01306-3.

## Background

Metastasis and therapy resistance are two leading causes of death for cancer patients, and epithelial–mesenchymal transition (EMT) is tightly associated with these two culprits [1, 2]. Among the molecular pathways that promote EMT to worsen the prognosis of cancer patients, TGF-β/Smad pathway plays a potent and prevalent role [3, 4]. Mechanistically, TGF-β binds its cell surface receptors, the type II and type I receptor complex, to induce the recruitment and phosphorylation of Smad2/3. The phosphorylated Smad2 and/or Smad3 forms an oligomer with Smad4 for translocation into the nucleus [5–7]. In the nucleus, Smad complex elicits multiple mechanisms to induce EMT. For instance, Smad activates the transcription of several EMT transcription factors, such as SNAI1, SNAI2, ZEB and Twist through direct or indirect mechanisms and increases the activity of Twist [8–11]. Smad also activates the expression of mesenchymal markers Fibronectin, Vimentin and Collagen αI [12, 13] and represses epithelial markers E-cadherin and occludin [14]. Through promoting EMT and EMT-associated malignant phenotypes such as invasion, metastasis, stem cell-like properties and immunosuppression/inflammation [3], TGF-β/Smad pathway functions as a key driver for cancer progression.

In addition to the potent tumor-promoting effects, TGF-β/Smad signaling also induces cytostatic effects, such as arresting cell cycle at G1 phase and inducing apoptosis [15]. By possessing both tumor-promoting and tumor-suppressive functions, the effects of TGF-β/Smad signaling on cancer are complex and often depend on the types and stages of cancer and the contexts of tumor cells and tumor microenvironment [16, 17]. For instance, TGF-β signaling usually prevents the generation of hyperplastic/premalignant lesions in normal tissues through its cytostatic effects. However, once tumors are progressed to an aggressive stage, the tumor-promoting effects become dominant. This functional switch of TGF-β/Smad signaling has been attributed to the activation of a number of oncogenic proteins that modulate Smad posttranslational modifications or Smad-controlled transcriptional programs [18]. For instance, c-Myc is known to counteract the growth inhibitory effect of TGF-β/Smad by reverting Smad-mediated transcriptional activation of CDK inhibitors *CDKN2B* and *CDKN1A*, also known as *p15INK4B* and *p21Cip1*, respectively [19–21]. Nevertheless, our understanding on the factors that dictate the net outcome of TGF-β/Smad signaling in cancer remains incomplete. Furthermore, several anti-TGF-β therapies showed poor clinical outcomes in cancer patients despite the success in the in vitro and preclinical models [22]. This points out the need for a better understanding of the cross talk between TGF-β pathway and other cancer pathways for stratifying patients that may benefit from the anti-TGF-β therapy or for designing more effective treatment regimens.

LncRNAs are known to elicit profound effects on cancer and can serve as prognostic/diagnostic biomarkers and therapeutic targets in many cancer types [23]. To date, lncRNAs have been shown to regulate a broad spectrum of cancer hallmarks and numerous cancer pathways [24]. TGF-β/Smad and c-Myc are among the “hot spots” for lncRNA-centered regulations. A large number of lncRNAs are reported to regulate c-Myc gene expression in *cis* or *trans*, influence on c-Myc protein stability or modulate c-Myc transcriptional activity [25, 26]. Similarly, a plethora of lncRNAs are linked to TGF-β/Smad signaling [3, 27]. These lncRNAs function as effectors to impact on a specific function of TGF-β signaling or act as regulators to modulate a specific step in the TGF-β/Smad signaling. Moreover, certain lncRNAs control a positive or negative feedback regulation of TGF-β/Smad signaling to amplify or dampen the signaling output, respectively. Despite the diverse modes of lncRNAs in regulating TGF-β/Smad signaling, lncRNAs that coordinate TGF-β signaling with other cancer pathways to alter the dichotomous roles of TGF-β in cancer have not been reported.

*Smyca* (Smad/Myc coactivator; also known as LOC284454) was originally identified as a p68-interacting RNA by RNA immunoprecipitation followed by high throughput sequencing. This 1.7 kb lncRNA is conserved only in primates and is generated by Drosha-mediated cleavage of a primary transcript for separating it from the precursor of miR-23a ~ 27a ~ 24–2 cluster [28]. Previous studies indicated that *Smyca* is upregulated in nasopharyngeal carcinoma and hepatocellular carcinoma (HCC) and associated with poor prognosis of these cancer types [29, 30]. Furthermore, serum level of *Smyca* is upregulated in nasopharyngeal carcinoma, oral cancer and thyroid cancer [31]. In this study, we identified *Smyca* based on its association with aggressive progression and poor prognosis of multiple cancer types. *Smyca* binds and activates Smad3 and Smad4 to enhance their interaction and promoter targeting. Additionally, *Smyca* binds c-Myc to enhance c-Myc-mediated transcription. Through potentiating both TGF-β/Smad and c-Myc pathways, *Smyca* promotes EMT and multiple EMT-associated malignant features, but avoids TGF-β-induced growth inhibition. Furthermore, *Smyca* acts through TGF-β and c-Myc signaling to synergize glycolysis. Consistent with the multifaceted tumor-promoting roles, targeting *Smyca* suppresses metastasis and sensitizes tumors to chemotherapy. Thus, *Smyca* is a lncRNA that turns the dichotomous roles of TGF-β toward tumor promotion and represents a potential therapeutic target for cancers with aberrant activation of TGF-β and/or c-Myc pathways.

## Materials and methods

### Cell culture and transfection

LM6 is a subclone of MDA-MB-231 cells derived from six-round enrichment of lung metastatic cells via an experimental metastasis model [32]. 293FT, MDA-MB-231, MDA-MB-468, Hs578T, MCF7, BT-549, BT-474, ZR75-1, LM6 cells and normal mammary epithelial cell line M10 were maintained in Dulbecco’s modified Eagle’s medium (DMEM) supplemented with 10% fetal bovine serum (FBS), 100 U/ml penicillin and 100 μg/ml streptomycin. The HCC cell lines Malaru and NTU-BL were grown in high-glucose DMEM supplemented with 10% FBS, 2 mM L-glutamine, 1 mM sodium pyruvate, 1 × non-essential amino acids, 100 U/ml penicillin and 100 μg/ml streptomycin. Transfection was performed using Lipofectamine 2000 reagent (Invitrogen) or TransIT-X2 transfection reagent (Mirus) according to the manufacturers’ instructions.

### Plasmids

The full-length *Smyca* cDNA was amplified by RT-PCR from HT29 cells and subcloned to pLAS5w, pRK5-Flag and pcDNA. To generate the *Smyca* (1-500), *Smyca* (501-1000), *Smyca* (1001-1500), *Smyca* (1501-1772) and *Smyca* (△1-500) constructs, the corresponding cDNA fragments were amplified via PCR and subcloned to pLAS5w and pcDNA. *Smyca* (△1001-1500) mutant was generated using the NEBuilder HiFi DNA Assembly Kit (New England Biolabs). To generate the Smad7 reporter construct, a DNA fragment corresponding to nucleotides − 584 to + 160 of the *Smad7* gene containing an SBE sequence (5’-GTCTAGAC) was amplified from the genomic DNA of MDA-MB-231 cells and inserted to pGL3-Basic plasmid. The SNAI2 reporter construct was described previously [33]. The 3TP-Luc and 4 × SBE-Luc constructs were obtained from Rik Derynck (University of California at San Francisco) and the SERPINE1-Luc construct was from Xin-Hua Feng (Zhejiang University, Zhejiang, China). The Myc-responsive reporter construct was purchased from Qiagen. The full-length cDNAs for Smad3 and Smad4 were amplified by PCR from pRK5F-Smad3 and pRK5F-Smad4 [34], respectively, and subcloned to pGEX4T-1 and pVL1392. To generate Smad3△MH1 and Smad4△MH1 mutants, Smad3 cDNA fragment containing nucleotides 409 to 1278 and Smad4 cDNA fragment containing nucleotides 427 to 1659 were amplified via PCR and subcloned to pVL1392. To generate GFP fusion constructs for full-length c-Myc, c-Myc TAD (1-150), c-Myc△TAD (151-439) and c-Myc△DBD (1–319), the corresponding cDNA fragments were amplified by PCR from pLAS3w-c-Myc and subcloned to pEGFP-N3.

### Antibodies and reagents

Antibodies used in this study were obtained from commercial sources and are described in Additional file 1: Table S1. Recombinant human proteins TGF-β, EGF and bFGF were obtained from R&D Systems, whereas c-Myc was purchased from Abcam. TβRI (type I TGF-β receptor) inhibitor SB431542, c-Myc inhibitor 10058-F4, cisplatin, doxorubicin (Dox), protamine sulfate and calf thymus DNA were purchased from Sigma-Aldrich (St Louis, MO). DOTAP, cholesterol and DSPE-PEG_2000_ were purchased from Avanti Polar Lipids (Alabaster, AL).

### RNA interference

*Smyca* shRNAs predicted via BLOCK-iT™ RNAi Designer (https://rnaidesigner.thermofisher.com/) were generated by Purigo Biotechnology, Inc. (Taiwan), and cloned to pLKO.1. siRNA targeting c-Myc was purchased from Dharmacon (Lafayette, CO, USA). Other shRNAs were from RNA Technology Platforms and Gene Manipulation Core Facility, Taiwan. The sequences of siRNAs and shRNAs are listed in Additional file 1: Table S2.

### Migration and invasion

Transwell migration and invasion assays were performed as described [35]. At the end of incubation, cells that had migrated onto the lower membrane surface were fixed with 4% formaldehyde, stained with 0.5% DAPI and photographed at least five random fields for each well under a Nikon fluorescence microscope. The migrated cells in each field were counted automatically via the “Analyze Particles” function of Image J.

### Spheroid formation

Cells were plated on 96-well ultra-low attachment plates at a tenfold serial dilution from 1 to 1000 cells per well and cultured in tumor sphere medium (DMEM/F12 medium supplemented with B27 (Invitrogen), 20 ng/ml EGF, 10 ng/ml bFGF and 5 μg/ml insulin (Invitrogen)). After 3 weeks, tumor spheres were imaged by an Olympus Scan^R high-content screen station (Olympus). Spheres with a diameter > 20 μm were scored and the percentage of wells containing tumor spheres was measured. The sphere-forming frequency of each group was analyzed via the extreme limiting dilution assay (ELDA) model (http://bioinf.wehi.edu.au/software/elda/).

### Flow cytometry

Cells were trypsinized by dissociation buffer (Invitrogen) and resuspended in blocking solution (Ca^2+^, Mg^2+^-free HBSS containing 2% goat serum). Next, cells were incubated with APC-conjugated anti-CD44 antibody (BD Bioscience) and PE-conjugated anti-CD24 antibody (BD Bioscience) for 1 h at 4 °C. After washes, the labeled cells were analyzed by a Beckman CytoFLEX flow cytometer. The breast cancer stem cell population (CD44^high^ and CD24^low/−^) was calculated via the CytExpert software.

### Quantitative RT-PCR (qRT-PCR)

Total RNAs were extracted by TRIZOL reagent (Invitrogen) and quantified by NanoDrop (Thermo Scientific). Reverse transcription was performed using iScript™ cDNA Synthesis Kit (Bio-Rad) according to the manufacturer’s instructions. Real-time PCR was performed on a LightCycler® 480 System with SYBR Green I Master Kit (Roche). GAPDH was used as an internal control. *Smyca* copy number was calculated by the equation of “Number of copies = Amount (ng) × 6.022 × 10^23^/Length (bp)x1 × 10^9^x660” in which “Amount” was determined by a “Ct value to amount” standard curve generated from known concentrations of a *Smyca*-containing plasmid. The PCR primers used in this study are listed in Additional file 1: Table S3.

### Luciferase assay

Cells transfected with the pGL3-based firefly luciferase construct together with the pRK5F-based Renilla luciferase plasmid were used for luciferase assay with the Dual-Glo Luciferase Reporter Assay System (Promega) followed by the manufacturer’s instructions. The firefly luciferase activity was normalized to that of Renilla luciferase activity.

### In situ hybridization

In situ hybridization was performed with the ViewRNA ISH Tissue 2-Plex Assay Kit (Thermo Fisher) according to the manufacturer’s instructions. Briefly, the paraformaldehyde-fixed cells were permeablized with protease and hybridized with the commercial *Smyca* tilling Type 1 probe set (Thermo Fisher, Catalog #VA1-3016541) at 40 °C for 2 h. After washing out the unlabeled probes, the hybridization signal was amplified by 2 steps of branch DNA hybridization at 40℃ for 40 min. These branch DNA amplifiers were then labeled by alkaline phosphatase, and fluorescence was developed by incubating with Fast Red substrates at 40 °C for 60 min. The slide was then counterstained with DAPI and examined by an Olympus FV3000RS inverted confocal microscope equipped with 60x/1.40 oil objective lens (Olympus Objective Lens, PlanApo N). Images were collected by an Olympus FV3000 FV31S-SW (v 2.40) software.

### Subcellular fractionation

Cells were trypsinized and passed through a 70 µm cell strainer (BD Falcon) to remove cell clumps. After centrifugation, the cell pellet was resuspended in 200 µl ice-cold cytoplasmic lysis buffer (10 mM Tris pH 7.5, 150 mM NaCl and 0.15% NP-40), chilled on ice for 5 min, transferred to a tube containing 500 µl ice-cold sucrose buffer (10 mM Tris pH 7.5, 150 mM NaCl and 24% sucrose) and centrifuged at 13,000 rpm for 10 min. The supernatant was collected as the cytoplasmic fraction. The pellet was resuspended in 200 µl of cytoplasmic lysis buffer without NP-40 and centrifuged again with 500 µl sucrose buffer. The pellet was washed and resuspended in 200 µl ice-cold glycerol buffer (20 mM Tris pH 7.5, 75 mM NaCl, 0.5 mM EDTA, 50% glycerol and 0.85 mM DTT) and then lysed by 200 µl of ice-cold nuclei lysis buffer (20 mM HEPES pH 7.5, 7.5 mM MgCl_2_, 0.2 mM EDTA, 300 mM NaCl, 1 M urea, 1% NP-40 and 1 mM DTT). The nuclear lysate was vortexed vigorously for 5 s, incubated on ice for 1 min and centrifuged at 14,000 rpm for 2 min. The supernatant was transferred to a fresh tube and saved as the nuclear fraction. 10% of the cytoplasmic and nuclear fractions were used for RNA extraction and qRT-PCR analysis.

### Western blot and immunoprecipitation

Cells were lysed with RIPA buffer containing 50 mM Tris pH 7.5, 150 mM NaCl, 1% NP-40, 0.5% deoxycholic acid, 0.1% SDS, 1 mM DTT, 1 mM phenylmethylsulphonyl fluoride (PMSF), 1 μg/ml aprotinin, 1 μg/ml leupeptin, 1 mM sodium vanadate, 4 mM sodium pyrophosphate and 20 mM NaF. Immunoprecipitation and Western blot using cell lysates with equal amounts of proteins were performed as described [36].

### In silico expression-based analysis and prediction

The RNA-seq data and survival information derived from patients of various cancer types were downloaded from The Cancer Genome Atlas (TCGA) via the UCSC Xena platform (http://xena.ucsc.edu) [37]. Microarray data from human breast, liver and colon cancer patients were retrieved from the NCBI’s Gene Expression Omnibus (GEO) database. The association between *Smyca* expression and cancer patient survival was conducted by Graphpad Prism using the Kaplan–Meier survival analysis along with the log-rank test. The correlation analysis for the expression of two genes was conducted by Pearson’s correlation analysis using the Python Statistical package scipy stats.

### RNA-sequencing (RNA-seq) analysis

Total RNAs were extracted by the NucleoSpin RNA Kit (Macherey-Nagel, Duren, Germany). Only RNA samples with a clear peak of 25–200 nt on the electrophenogram (small RNA fraction) and an RNA integrity number of at least 8 were used for the subsequent library construction and sequencing. After removing rRNAs from the RNA samples, RNA libraries were generated by the Agilent Sure Select Strand Specific RNA Library Prep Kit (Illumina). Next-generation sequencing was performed in HiSeq4000 (Illumina) for 150 bp paired-end reads by Novogene (Biotools). Reads were mapped against the human reference genome (GRCh38/hg38) using TopHat v2.0.12 to generate align reads for each sample. The align reads were counted and normalized to per million bases by HTSeq to obtain gene abundance. Differentially expressed genes (DEGs) were determined using DESeq v1.10.1.

### Pathway and gene set enrichment analyses

The DEGs identified by RNA-seq were subjected to the Ingenuity Pathway Analysis (IPA, Qiagen) or a web-based gene set analysis toolkit WebGestalt, 2019 (http://www.webgestalt.org/). To confirm the enrichment between *Smyca*-induced signature and published malignancy-related signatures from the MSigDB database, *Smyca*-induced transcriptome was submitted to the Gene Set Enrichment Analysis (GSEA) v4.1.0 Java Web Start. Gene sets with a false discovery rate (FDR) < 0.05 by comparing the enrichment score to the enrichment results generated from 1000 random permutations were considered as statistically significant.

### Chromatin immunoprecipitation (ChIP)

Cells were treated with 1% formaldehyde for 15 min for cross-linking the chromatins followed by quenched with 125 mM glycine for 5 min. Next, cells were lysed with ChIP lysis buffer (3 mM HEPES pH 7.5, 140 mM NaCl, 1 mM EDTA, 0.5 mM EGTA, 1% Triton X-100, 0.1% sodium deoxycholate, 0.1% SDS, 0.5% N-lauroylsarcosine, 1 mM PMSF, 1 μg/ml aprotinin, 1 μg/ml leupeptin, 1 mM sodium vanadate, 4 mM sodium pyrophosphate and 20 mM NaF) and sonicated to achieve the majority of DNA fragments within 200–500 bp. The lysate was precleared with Protein A and incubated with the desired antibodies at 4 °C for overnight, followed by the addition of 100 μl Protein-A magnetic beads with rotation for 1 h. The immunocomplexes were washed 4 times with ChIP wash buffer (50 mM HEPES pH 7.5, 1 mM EDTA, 1% NP-40, 500 mM LiCl, 0.7% sodium deoxycholate, 1 mM PMSF, 1 μg/ml aprotinin, 1 μg/ml leupeptin, 1 mM sodium vanadate, 4 mM sodium pyrophosphate and 20 mM NaF), followed by TE buffer (10 mM Tris pH 8.0 and 1 mM EDTA) containing 50 mM NaCl. The protein–DNA complex was eluted twice from beads with 150 μl elution buffer (1% SDS, 200 mM NaCl, 100 µg/ml RNase A, 100 U/ml RNase H in TE buffer) at 65 °C for 20 min. The immunoprecipitated DNA fragments were incubated with 1 mg/ml proteinase K at 65 °C for 2 h to remove the cross-linked proteins, purified by a PCR purification kit (Qiagen) and analyzed by quantitative PCR (qPCR). The sequences of qPCR primers are listed in Additional file 1: Table S3.

### In silico analysis of ChIP-seq data

The Smad2/3 ChIP-seq data derived from TGF-β-treated Hs578T and BT-549 cells were downloaded from GSE83788. The aligned BAM files of ChIP-seq datasets were then subjected to MACS2 (Galaxy Version 2.1.1.20160309.6) to identify the Smad2/3 binding sites with a threshold of FDR < 0.05. The binding sites were mapped to the nearest genes by Galaxy platform (https://usegalaxy.org/) using the definition of promoter region as a segment between 2 kb upstream and 10 kb downstream of the transcriptional starting site (12 kb in length) in the human reference sequence assembly (NCBI Build 37/hg19, February 2009). The Smad2/3 occupied genes were then converted to “Gene Symbol” by BioDBnet (https://biodbnet-abcc.ncifcrf.gov/).

### Chromatin isolation by RNA purification (ChIRP)

ChIRP was performed by adapting a protocol described previously [38]. Briefly, tiling 20-mer antisense oligonucleotide probes targeting *Smyca* and the negative control probes for *lacZ* RNA were designed using the online probe designer at singlemoleculefish.com (http://www.singlemoleculefish.com/designer.html), synthesized and biotinylated by Genomics, Inc. The sequences of these probes are listed in Additional file 1: Table S4. Cells were treated with 1.25% glutaraldehyde for 15 min and then with 125 mM glycine for 5 min. The cross-linked chromatins were sheared into 200–500 bp by sonication and hybridized with each of the probe sets, followed by streptavidin magnetic beads capturing and wash/elution steps. The ChIRP captured chromatins were treated with Protease/RNase and analyzed by qPCR. The sequences of qPCR primers are listed in Additional file 1: Table S3.

### RNA pull-down analysis

Biotin-labeled RNAs were generated by in vitro transcription using the AmpliScribe T7-Flash Biotin-RNA Transcription Kit (Epicentre Biotechnologies) and purified by the NucleoSpin RNA Kit (Macherey-Nagel, Duren, Germany). To form the proper secondary structure, 30 pmol biotinylated RNA was heated to 90 °C in the RNA structure buffer (10 mM Tris pH 7.0, 100 mM KCl and 10 mM MgCl_2_) for 2 min, chilled on ice for 2 min and incubated at room temperature for 20 min. The RNA was mixed with nuclear extracts or purified proteins and incubated at room temperature for 1 h, followed by incubating with Streptavidin magnetic beads (GE Healthcare) at room temperature for 1 h. After washes, the pull-down complexes were analyzed by Western blot. Alternatively, the folded RNA was incubated with recombinant protein conjugated on beads at room temperature for 1 h. After washes, the pull-down RNA was extracted by TRIZOL reagent and analyzed by qRT-PCR.

For preparing nuclear extracts, cells were harvested by 2 ml PBS and mixed with 8 ml nuclear isolation buffer (0.32 M sucrose, 10 mM Tris pH 7.5, 5 mM MgCl_2_ and 1% Triton X-100) on ice for 20 min with frequent mixing. After centrifugation at 2,500 × *g* for 15 min, the pellet was resuspended with 1 ml ice-cold RIP buffer (150 mM KCl, 25 mM Tris pH 7.4, 5 mM EDTA, 0.5% NP-40, 0.5 mM DTT, 1 mM PMSF, 1 μg/ml aprotinin, 1 μg/ml leupeptin, 1 mM sodium vanadate, 4 mM sodium pyrophosphate, 20 mM NaF and 100 U/ml SUPERaseIN). The nuclear lysate was sonicated using a Qsonica Q700 system according to the manufacturer’s protocol, followed by centrifugation at 13,000 rpm for 20 min. The supernatant was recovered as the nuclear extract.

### RNA immunoprecipitation (RIP)

Cells were lysed with polysome lysis buffer containing 15 mM Tris pH 7.5, 300 mM NaCl, 1% Triton X-100, 1 mM DTT, 1 mM PMSF, 1 μg/ml aprotinin, 1 μg/ml leupeptin, 1 mM sodium vanadate, 4 mM sodium pyrophosphate, 20 mM NaF and 100 U/ml SUPERaseIN. The lysates were precleared with Protein-A magnetic beads (Millipore, Bedford, MA, USA) at 4 °C for 60 min. After adding 2 μg antibody into the precleared lysates, the immunoprecipitated protein-RNA complex was captured by Protein-A magnetic beads at 4 °C for 2 h. For experiment involving sequential immunoprecipitations, the bound proteins were eluted twice with 150 μg/ml M2 peptide in TBS buffer (50 mM Tris pH 7.5 and 150 mM NaCl). The eluent was diluted to 1 ml with the adjustment of salts and detergent to 50 mM Tris pH 7.5, 150 mM NaCl, 1% NP-40, 5% glycerol and 5 mM EDTA, followed by the second immunoprecipitation. The immunoprecipitated proteins and coprecipitated RNAs were extracted from the beads with sample buffer and TRIZOL reagent and analyzed by qRT-PCR and immunoprecipitation, respectively.

### Cell proliferation and viability assays

To determine the proliferation rate, cells were seeded on 96-well plates at a density of 2000 cells/well, cultured overnight and treated with various inhibitors. Alternatively, transfected cells were seeded at a density of 8000 cells/well. Then, cells were pulse labeled with 10 μM BrdU for 2 h. After fixation, BrdU incorporation was determined by the BrdU Cell Proliferation Assay Kit (Merck Millipore) according to the manufacturer's instructions. To test cell viability in response to chemotherapeutic agents, cells were seeded at a density of 2000 cells/well on 96-well plates. After attachment, cells were cultured in medium containing Dox or cisplatin for 2 or 3 days, respectively. Cell viability was determined by incubating with 0.4 mg/ml methyl thiazolyl diphenyl tetrazolium bromide (MTT, Sigma-Aldrich) for 2 h, followed by cell lysis with DMSO and absorbance measurement at 590 nm.

### Measurement of glucose and lactate levels

Cells were seeded on 6-well plates at a density of 2 × 10^5^ cells/well and treated with various inhibitors. The conditioned media were harvested at 0 and 24 h (for measuring glucose) or 0 and 48 h (for measuring lactate) post-treatment. The concentrations of glucose and lactate were determined by commercial ELISA-based kits (BioVision, Inc.) according to the manufacturer's instructions.

### Measurement of extracellular acidification rate (ECAR)

ECAR was determine by the Seahorse Extracellular Flux analyzer (XFe24, Seahorse Bioscience, Billerica, MA, USA) with the Seahorse XF Glycolysis Stress Test Kit according to the manufacturer’s instructions. Briefly, cells were seeded at a density of 40,000 cells/well in 24-well plates and allow to attach. Three hours later, the cells were treated with various pathway inhibitors for 24 h. The glycolytic metabolic profiles were determined by sequential injections of glucose (10 mM), oligomycin (1 uM) and 2-deoxy-D-glucose (75 mM). The Seahorse Wave 2.3 software was used for data analyses, and ECAR was expressed in mpH/min. The maximum glucose response was referred to as glycolysis, whereas the glycolytic reserve was calculated as: maximum oligomycin response—maximum glucose response.

### Preparation of PEGylated liposome–polycation DNA nanoparticle (LPD-NP) formulations

LPD-NPs were prepared according to a previously method [39, 40] with slight modifications. Briefly, liposomes composed of DOTAP and cholesterol (1:1 molar ratio) were prepared by thin film hydration followed by sonication for 3 min (5 s on/5 s off, Amp 30%). To prepare LPD-NPs, 22 µl of protamine (2 mg/ml), 120 µl of deionized water, and 24 µl of 1:1 weight ratio of gapmer (Qiagen, 2 mg/ml) and calf thymus DNA (2 mg/ml) were mixed and kept at room temperature for 10 min before adding 60 µl of liposome (20 mmol/l). LPD-NPs were stood at room temperature for 10 min before the addition of DSPE-PEG. LPD-NPs were then mixed with 33.6 µl of DSPE-PEG_2000_ (10 mg/ml) and kept at 50–60 °C for 10 min. The sequences of gapmers are listed in Additional file 1: Table S2.

### Animal studies

For experimental metastasis model, MDA-MB-231 cells tagged with luciferase were resuspended (1 × 10^6^ cells/0.1 ml HBSS) and injected into the tail vein of 7-week-old female NOD/SCID (NOD.CB17-*Prkdc*^*scid*^/NcrCrlBltw) mice (BioLASCO Taiwan Co., Taipei, Taiwan) by a 27-gauge needle. Lung metastasis was monitored by bioluminescence imaging using the PerkinElmer In Vivo Imaging System (Waltham, MA, USA). The mice were then killed for histological analysis of the lung.

To monitor chemoresistance, 2 × 10^6^ MDA-MB-231 cells were orthotopically injected into the fat pad of 7-week-old female Nu/Nu (Bltw:NU-*Foxn1nu)* mice (BioLASCO Taiwan Co., Taipei, Taiwan). When tumors grew to about 30 mm^3^ in volume, Dox (6 mg/kg) diluted with 0.9% NaCl was administered via intraperitoneal injection every week. The sizes of tumors were measured every 3 or 4 days, and their volumes were calculated using the equation mm^3^ = 1/2 × length (mm) × (width (mm))^2^.

To evaluate the anti-tumor effect of LPD-NPs containing *Smyca* gapmer ASO, LM6 tumor-bearing NOD/SCID mice were injected intratumorally with 30 µg LPD-NPs and intraperitoneally with Dox (6 mg/kg) every week. The sizes of tumors were measured every 3 or 4 days. At the end of experiment, the tumors were removed to visualize their sizes. To evaluate the anti-metastasis effect of LPD-NPs containing *Smyca* gapmer ASO, LM6 tumor-bearing NOD/SCID mice were intratumorally injected with 30 µg LPD-NPs every week. Lung metastasis was monitored by bioluminescence imaging. All mouse experiments were conducted with the approval from the Experimental Animal Committees of Academia Sinica and Taipei Medical University.

### Human specimens

Snap-frozen primary breast cancer tissues stored in liquid nitrogen were obtained from Taipei Medical University BioBank and mRNAs extracted from HCC tissues and paired noncancerous adjacent tissues were obtained from National Health Research Institutes BioBank. Written informed consents were obtained from all patients. The research design, study protocols and information security were approved by the Institutional Review Boards of Taipei Medical University and Academia Sinica.

## Results

### *Smyca* high expression correlates with poor prognosis and aggressive progression of multiple cancer types

In an attempt to identify cancer-relevant lncRNAs, we searched for lncRNAs associated with adverse prognosis of cancer. By querying TCGA data sets from a variety of cancer types, we found that *Smyca* high expression correlated with poor overall survival and disease-free survival of several cancer types, including kidney clear cell carcinoma, lower grade glioma, adrenocortical cancer and mesothelioma (Additional file 1: Fig. S1A, B). Furthermore, the association of *Smyca* high expression with poor overall survival was also observed from two breast cancer cohorts and one colon cancer cohort downloaded from the GEO database as well as an in house HCC cohort (Additional file 1: Fig. S1C–F). In the HCC cohort, although *Smyca* expression did not differ significantly between tumor and adjacent non-tumor tissues, its high expression was modestly but significantly associated with higher tumor stages and invasive phenotypes (Additional file 1: Fig. S1G–I). Similar associations were observed from an in house breast cancer cohort (Additional file 1: Fig. S1J, K). In this cohort, *Smyca* expression was higher in the basal-like subtype, compared with the less malignant HER2-enriched and luminal subtypes (Additional file 1: Fig. S1L). These findings collectively support *Smyca* as a prognostic marker for several cancer types and suggest its role in tumor promotion.

### *Smyca* promotes EMT, cancer stemness, migration and invasion

**Fig. 1:**
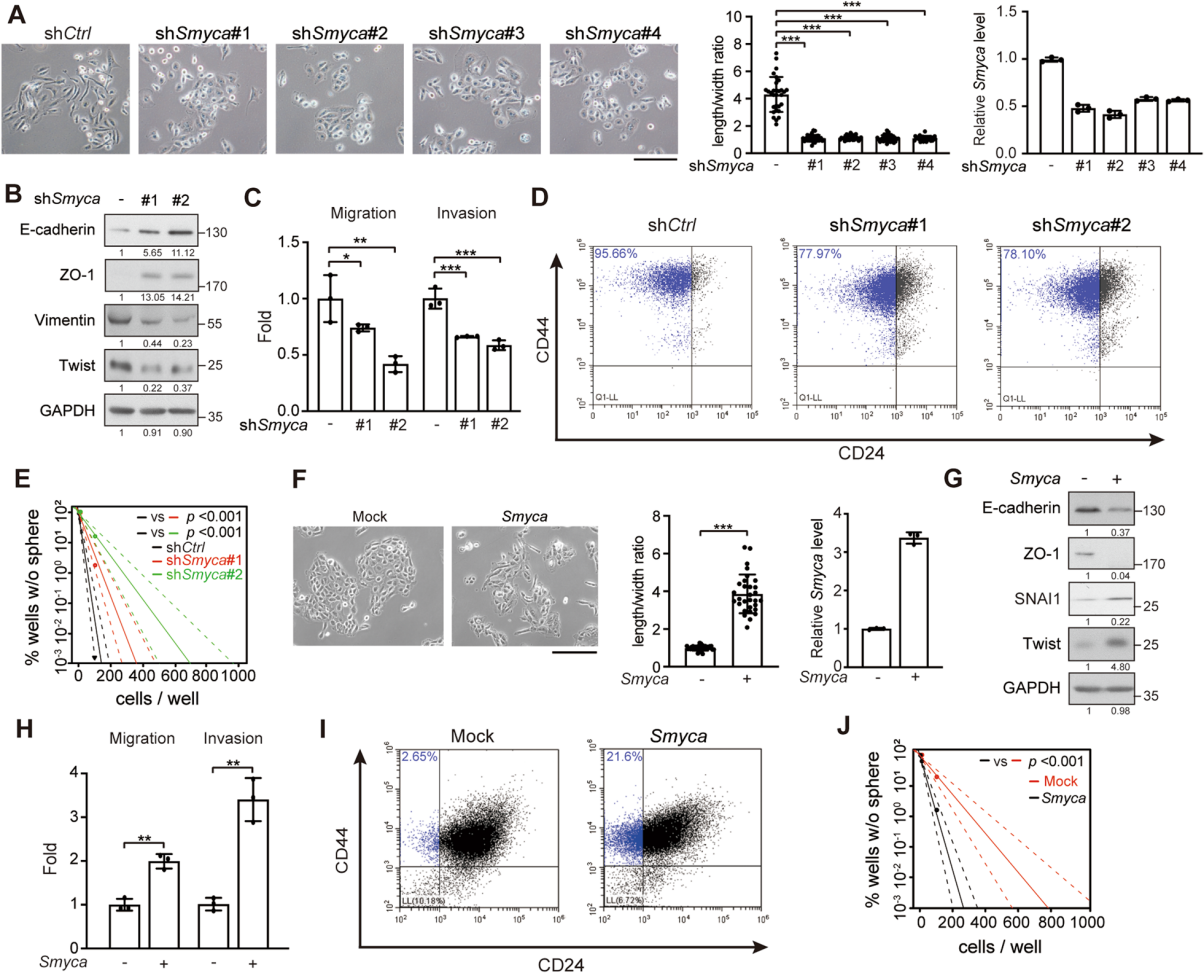
*Smyca* promotes EMT, migration, invasion and stemness. (**A**) Morphological assessment of MDA-MB-231 cells stably expressing four different *Smyca* shRNAs. Bar, 100 μm. Cell length/width ratios and *Smyca* expression levels (normalized to the control group) are shown in the middle and right panels, respectively. Data are mean ± SD, *n* = 30 (middle) or 3 (right). *P* values are determined by one-way ANOVA with Tukey’s post hoc test, *** *P* < 0.001. (**B**, **G**) Western blot analysis of EMT markers in MDA-MB-231 cells stably expressing *Smyca* shRNAs (**B**) or M10 cells stably overexpressing *Smyca* (**G**). The amounts of each protein in relation to the control cells are indicated under the bands. (**C**, **H**) Migration and invasion assays of MDA-MB-231 cells stably expressing *Smyca* shRNAs (**C**) or M10 cells stably overexpressing *Smyca* (**H**). Data are mean ± SD from three independent experiments. *P* values are determined by one-way ANOVA with Tukey’s post hoc test (**C**) or unpaired *t* test (**H**), *P < 0.05, **P < 0.01, *** *P* < 0.001. (**D**, **I**) Flow cytometry analysis of the expression of breast cancer stem cell markers in MDA-MB-231 cells stably expressing *Smyca* shRNAs (**D**) or M10 cells stably overexpressing *Smyca* (**I**). Stem cell populations are marked by blue and the percentages are indicated. (**E**, **J**) Sphere formation assay of MDA-MB-231 cells stably expressing *Smyca* shRNAs (**E**) or M10 cells stably overexpressing *Smyca* (**J**). Solid and dashed lines are derived from means and standard deviations, respectively. Data are mean ± SD from three independent experiments. *P* values are determined by Chi-square test. (**F**) Morphological assessment of M10 cells stably overexpressing *Smyca*. Bar, 100 μm. Cell length/width ratios and *Smyca* expression levels (normalized to the control group) are shown in the middle and right panels, respectively. Data are mean ± SD, *n* = 30 (middle) or 3 (right). *P* values are determined by unpaired *t* test, *** *P* < 0.001

In line with the findings derived from breast cancer patients, *Smyca* showed higher expression levels in mesenchymal/basal-like than epithelial-like breast cancer cell lines (Additional file 1: Fig. S2A). We therefore investigated whether *Smyca* promotes EMT, a feature of mesenchymal/basal-like subtype. Four shRNAs were individually introduced to mesenchymal/basal-like breast cancer cell line MDA-MB-231. These shRNAs reduced *Smyca* expression without affecting the expression of miRNAs in the miR-23a ~ 27a ~ 24–2 cluster (Fig. 1A and Additional file 1: Fig. S2B). *Smyca* knockdown switched the mesenchymal morphology of MDA-MB-231 cells to epithelial, as indicated by the reduction in cell length/width ratio (Fig. 1A). Furthermore, *Smyca* knockdown elevated the expression of epithelial markers E-cadherin and ZO-1 and reduced the mesenchymal markers Vimentin and Twist (Fig. 1B). These findings are consistent with an induction of mesenchymal-to-epithelial transition (MET). *Smyca* knockdown in another mesenchymal/basal-like breast cancer cell line Hs578T similarly upregulated epithelial markers and downregulated mesenchymal markers (Additional file 1: Fig. S2C). In line with the MET induction, *Smyca* knockdown in MDA-MB-231 cells inhibited migration and invasion (Fig. 1C). Next, we evaluated the effect of *Smyca* on cancer stemness, a feature tightly linked to EMT. By monitoring the breast cancer stem-like cell markers CD44^high^/CD24^low/−^, we found a high stem-like population in MDA-MB-231 cells, consistent with previous reports [41, 42]. This stem-like population was reduced by *Smyca* knockdown (Fig. 1D). Furthermore, *Smyca* knockdown decreased the ability of MDA-MB-231 cells to form mammary spheres (Fig. 1E). In the reciprocal sets of experiments, we chose to overexpress *Smyca* in mammary epithelial cell M10 and epithelial-like breast cancer cell line MCF7, which expressed lower levels of *Smyca* comparing to MDA-MB-231 cells (Additional file 1: Fig. S2A). We found that *Smyca* overexpression in these cells promoted EMT (Fig. 1F, G and Additional file 1: Fig. S2D). *Smyca* overexpression in M10 cells also promoted migration and invasion and enhanced stemness features (Fig. 1H–J). Notably, *Smyca* overexpression or knockdown did not affect cell proliferation and survival (Additional file 1: Fig. S2E–H). Thus, our study revealed a critical role of *Smyca* in mediating EMT and EMT-associated malignant features, such as migration, invasion and tumor stemness.

### ***Smyca*** promotes TGF-β/Smad signaling

**Fig. 2:**
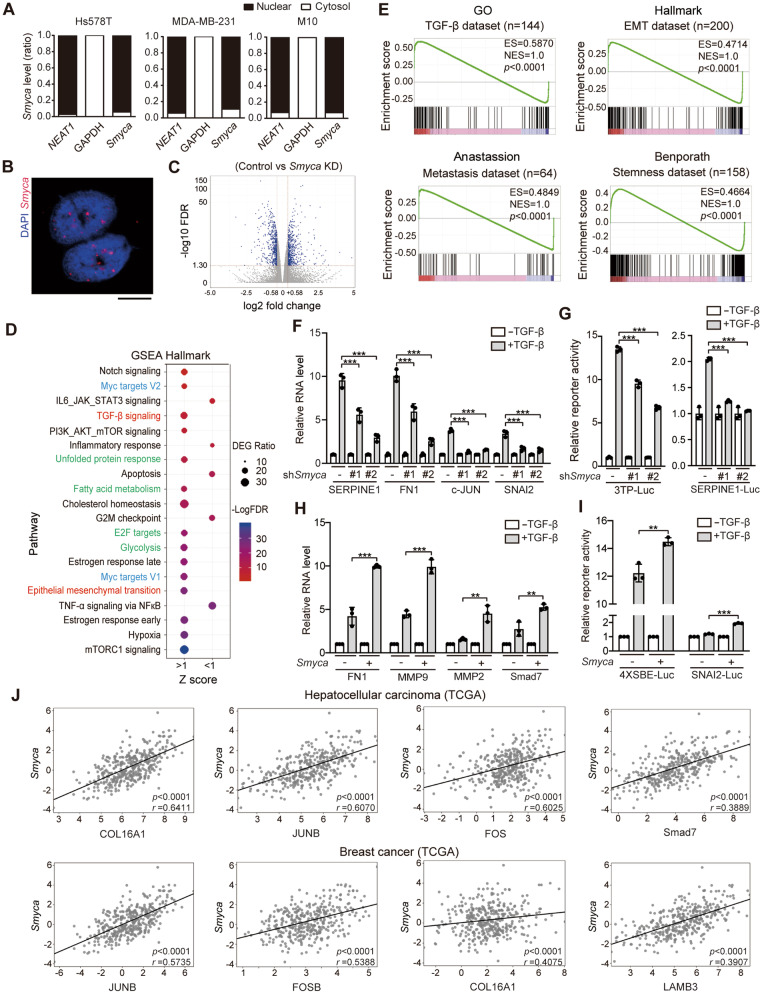
*Smyca* enhances TGF-β signaling. (**A**) qRT-PCR analysis of the ratios of nuclear and cytoplasmic *Smyca* from indicated cells. *NEAT1* and *GAPDH* were used as controls. (**B**) Representative image for *Smyca* subcellular distribution analyzed by in situ hybridization on MDA-MB-231 cells. Bar, 10 μm. (**C**) Comparison of RNA-seq data derived from MDA-MB-231 cells expressing control shRNA and *Smyca* shRNA #1. DEGs are marked by blue dots. (**D**) GSEA Hallmark Pathway analysis of DEGs shown in (**C**). The top enriched and depleted hallmarks are shown by the order of FDR (bottom to top). (**E**) Representative GSEA plots for the match of *Smyca* signature with the indicated signatures. Enrichment score (ES) and normalized enrichment score (NES) are indicated. The full set of GSEA data is shown in Additional file 1: Fig. S3A. (**F**, **H**) qRT-PCR analysis of indicted genes in MDA-MB-231 cells stably expressing *Smyca* shRNAs (**F**) or M10 cells stably expressing *Smyca* (**H**) and treated with or without 5 ng/ml TGF-β for 24 h. Data are normalized with that of untreated group in each cell. (**G**, **I**) Luciferase reporter assay on MDA-MB-231 cells stably expressing *Smyca* shRNAs (**G**) or M10 cells stably expressing *Smyca* (**I**), transfected with indicated reporters and treated with or without 5 ng/ml TGF-β for 24 h. Data in (**F**), (**G**), (**H**), and (**I**) are normalized with that of untreated control and expressed as mean ± SD from three independent experiments. *P* values are determined by one-way ANOVA with Tukey’s post hoc test (**F**, **G**) or unpaired *t* test (**H**, **I**), **P < 0.01, ***P < 0.001. (**J**) Representative correlation plots of *Smyca* expression with the expression of indicated TGF-β target genes by analyzing HCC or breast cancer data sets from TCGA (*n* = 369 for HCC and 1099 for breast cancer). Pearson’s coefficients and *P* values are indicated. Additional correlative data are shown in Additional file 1: Fig. S3F

Next, we explored the mechanism by which *Smyca* potentiates EMT. Cell fractionation analysis and fluorescence in situ hybridization assay revealed that *Smyca* was mainly distributed to the nucleus (Fig. 2A, B). Since nuclear lncRNAs often regulate gene expression, we determined the impact of *Smyca* on transcriptome by RNA-seq analysis of MDA-MB-231 cells expressing control or *Smyca* shRNA. DEGs were defined with the criteria: control/*Smyca* knockdown ≥ 1.5 or ≤ 0.66 and FDR < 0.05 and a total of 2201 DEGs were recovered in three biological repeats (Fig. 2C). GSEA Hallmark Pathway analysis of these DEGs revealed that EMT and TGF-β signaling were among the upregulated hallmarks (Fig. 2D, red). Furthermore, GSEA analysis found that *Smyca*-induced transcriptome correlated significantly and positively with TGF-β, stemness, metastasis and EMT signatures from multiple sources (Fig. 2E and Additional file 1: Fig. S3A). Comparison of *Smyca*-induced DEGs with the Smad2/3 ChIP-seq data derived from Hs578T or BT-549 cells [43] revealed a subset of overlapped genes (Additional file 1: Fig. S3B and Table S5). These findings prompted us to investigate the impact of *Smyca* on TGF-β/Smad signaling. Importantly, *Smyca* knockdown in MDA-MB-231 cells diminished TGF-β-induced expression of multiple downstream genes, such as *FN1* (Fibronectin 1), *SERPINE1* (encoding PAI-1), *c-JUN* and *SNAI2* and TGF-β-mediated transcriptional activation of Smad-responsive reporters 3TP-Luc and SERPINE1-Luc (Fig. 2F, G). *Smyca* knockdown in Hs578T cells similarly attenuated TGF-β-induced expression of downstream genes and activation of Smad-responsive reporters 4 × SBE-Luc and SERPINE1-Luc (Additional file 1: Fig. S3C, D). A similar reduction in TGF-β-induced 4 × SBE-Luc reporter activity was found by *Smyca* knockdown in HCC cell line Malaru (Additional file 1: Fig. S3E), which expressed a higher level of *Smyca* than another HCC cell line NTU-BL (Additional file 1: Fig. S2A). Conversely, *Smyca* overexpression in M10 cells increased TGF-β-induced downstream gene expression and Smad-responsive reporter activities (Fig. 2H, I). These data identify *Smyca* as a positive regulator of TGF-β/Smad signaling, which is in line with its function in promoting EMT and EMT-associated tumor malignancies.

To validate the clinical relevance of *Smyca* to TGF-β-induced transcriptional program, we queried RNA-seq data from TCGA data sets. Remarkably, *Smyca* expression correlated positively (Pearson’s correlation *r* > 0.3) with the expression of a large set of TGF-β/Smad target genes in the tumor tissues of HCC and breast cancer patients (Fig. 2J and Additional file 1: Fig. S3F). Similar findings were obtained by analyzing the microarray data of breast cancer and HCC patients from the GEO data sets (Additional file 1: Fig. S3G). Importantly, most of these genes are of tumor-promoting functions and contribute to invasion and metastasis. GO analysis of this set of genes revealed that response to TGF-β, cell migration/chemotaxis, cell adhesion mediated by integrin signaling and collagen metabolic process are among the enriched GO terms (Additional file 1: Fig. S3H). Thus, these data revealed the association of *Smyca* with TGF-β-induced transcription of a set of tumor-promoting genes in human tumor tissues, highlighting the clinical relevance of *Smyca*-promoted TGF-β signaling to tumor progression.

### *Smyca* enhances Smad3/Smad4 complex formation and recruitment to its target promoters

**Fig. 3:**
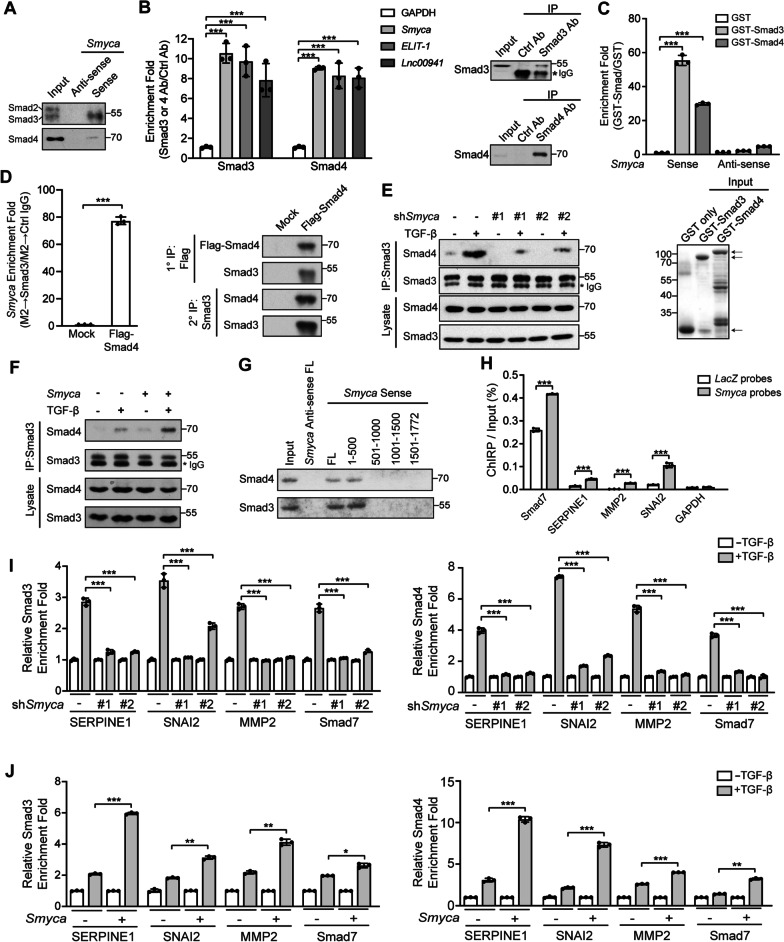
*Smyca* enhances Smad3/Smad4 complex formation and promoter recruitment. (**A**, **G**) RNA pull-down assay using MDA-MB-231 nuclear extracts and biotinylated sense or antisense *Smyca* or *Smyca* deletion fragments. Antibodies that recognize Smad2/3 and Smad3 only were used in (**A**) and (**G**), respectively. (**B**) RIP analysis for the enrichment of indicated lncRNAs in Smad3 or Smad4 immunoprecipitates derived from MDA-MB-231 cells. Data are normalized with that from the control antibody. The presence of Smad3 or Smad4 in the immunoprecipitates is shown on the right. (**C**) Bacterially purified GST-Smad3 or GST-Smad4 bound on beads was incubated with sense or antisense *Smyca*. The bound *Smyca* was analyzed by qRT-PCR and normalized with that from the GST only group. The input GST fusion proteins are shown on the bottom and marked by arrows. (**D**) Smad4-associated complex was immunoprecipitated from MDA-MB-231 cells transfected with Flag-Smad4 and treated with 5 ng/ml TGF-β for 2 h. The immunocomplex was eluted and further precipitated with anti-Smad3 antibody to isolate the Smad3/Smad4 complex. *Smyca* enrichment in this complex was analyzed by qRT-PCR. (**E**, **F**) Immunoprecipitation analysis of Smad3 and Smad4 interaction in MDA-MB-231 cells stably expressing *Smyca* shRNAs (**E**) or M10 cells stably expressing *Smyca* (**F**) and treated with or without 5 ng/ml TGF-β for 1 h. (**H**) ChIRP assay for *Smyca* occupancy on the indicated Smad target loci. *GAPDH* was used as a control. Tilling biotinylated oligonucleotides complementary to *LacZ* or *Smyca* were used to pull down the RNA-associated chromatins from MDA-MB-231 cells treated with 5 ng/ml TGF-β for 2 h, followed by qRT-PCR analysis. Data are normalized with the inputs. (**I**, **J**) ChIP analysis of the recruitment of Smad3 or Smad4 to the indicated promoters in MDA-MB-231 cells stably expressing *Smyca* shRNAs (**I**) or *Smyca* (**J**) and treated with or without 5 ng/ml TGF-β for 2 h. The enrichment folds are normalized with that from untreated group. Data in (**B**), (**C**), (**D**), (**H**), (**I**), and (**J**) are mean ± SD, *n* = 3. *P* values are determined by one-way ANOVA with Tukey’s post hoc test (**B**, **C**, **I**) or unpaired *t* test (**D**, **H**, **J**), *P < 0.05, **P < 0.01, ***P < 0.001

We next investigated how *Smyca* promotes TGF-β signaling. Given the enrichment of *Smyca* in the nucleus, we explored its impact on Smads. RNA pull-down assay by incubating in vitro transcribed *Smyca* with MDA-MB-231 nuclear lysates revealed that *Smyca* interacted specifically with Smad3 and Smad4, but not Smad2 (Fig. 3A). RIP analysis further detected a significant enrichment of *Smyca* in the Smad3 and Smad4 immunoprecipitates, and the enrichment folds were comparable to that of other Smad-binding lncRNAs, such as *ELIT-1* and *Lnc00941* (Fig. 3B). To determine whether *Smyca* binds Smad3 and Smad4 directly, bacterially purified GST-Smad3 or GST-Smad4 was incubated with in vitro transcribed *Smyca* or its antisense RNA. We observed a robust enrichment of *Smyca*, but not its antisense RNA, in beads containing GST-Smad3 or GST-Smad4, compared with GST alone (Fig. 3C). To interrogate whether *Smyca*, Smad3 and Smad4 form a tertiary complex in vivo, we introduced Flag-Smad4 into MDA-MB-231 cells, stimulated cells with TGF-β, and performed two-step immunoprecipitation analysis to isolate the Smad3/Smad4 complex. We found that *Smyca* was highly enriched in the Smad3/Smad4 complex (Fig. 3D). Because *Smyca* assembles a tripartite complex with Smad3 and Smad4, we determined whether *Smyca* enhances the association between the two Smads. *Smyca* knockdown in MDA-MB-231 cells attenuated TGF-β-induced Smad3/Smad4 association, without affecting the expression of Smad3 and Smad4 and the phosphorylation of Smad3 (Fig. 3E and Additional file 1: Fig. S4A, B). Conversely, *Smyca* overexpression in M10 or MDA-MB-231 cells enhanced TGF-β-induced Smad3/Smad4 complex formation (Fig. 3F and Additional file 1: Fig. S4C). To investigate the structural determinants for the association among *Smyca*, Smad3 and Smad4, we mapped their interaction regions. Using a panel of deletion mutants, we found that *Smyca* (1–500) segment bound Smad3 and Smad4 as efficiently as the full-length *Smyca* in a RNA pull-down assay, whereas other fragments bound neither Smad3 nor Smad4 (Fig. 3G). Accordingly, removal of the 1–500 segment abrogated the potentiating effects of *Smyca* on TGF-β-induced Smad3/Smad4 association and Smad-responsive reporter activity (Additional file 1: Fig. S4C, D). Deletion mapping analysis further showed a critical role of the MH1 domains of both Smad3 and Smad4 in their interactions with *Smyca* (Additional file 1: Fig. S4E). These findings support that *Smyca* (1-500) segment binds the MH1 domains of Smad3 and Smad4, which do not overlap with the MH2 domains responsible for the p-Smad3/Smad4 interaction [44]. Thus, *Smyca* functions as a scaffold to provide an additional binding surface for enhancing the association of Smad3 with Smad4.

To determine whether *Smyca* acts directly on the Smad3/Smad4 target chromatins, we conducted ChIRP assay using a set of probes complementary to the *Smyca* sequence for pulling down *Smyca* from TGF-β-stimulated MDA-MB-231 cells. Subsequent qPCR analysis demonstrated the recruitment of *Smyca* to promoters of a set of Smad targets (Fig. 3H). Furthermore, ChIP assay showed that *Smyca* knockdown reduced the recruitment of Smad3 and Smad4 to the promoter regions of these Smad targets, whereas *Smyca* overexpression elicited an opposite effect (Fig. 3I, J). Thus, our findings suggest that the *Smyca*/Smad3/4 complex acts on chromatins to promote the transcription of Smad target genes.

### ***Smyca*** governs a positive feedback regulation of TGF-β signaling

**Fig. 4:**
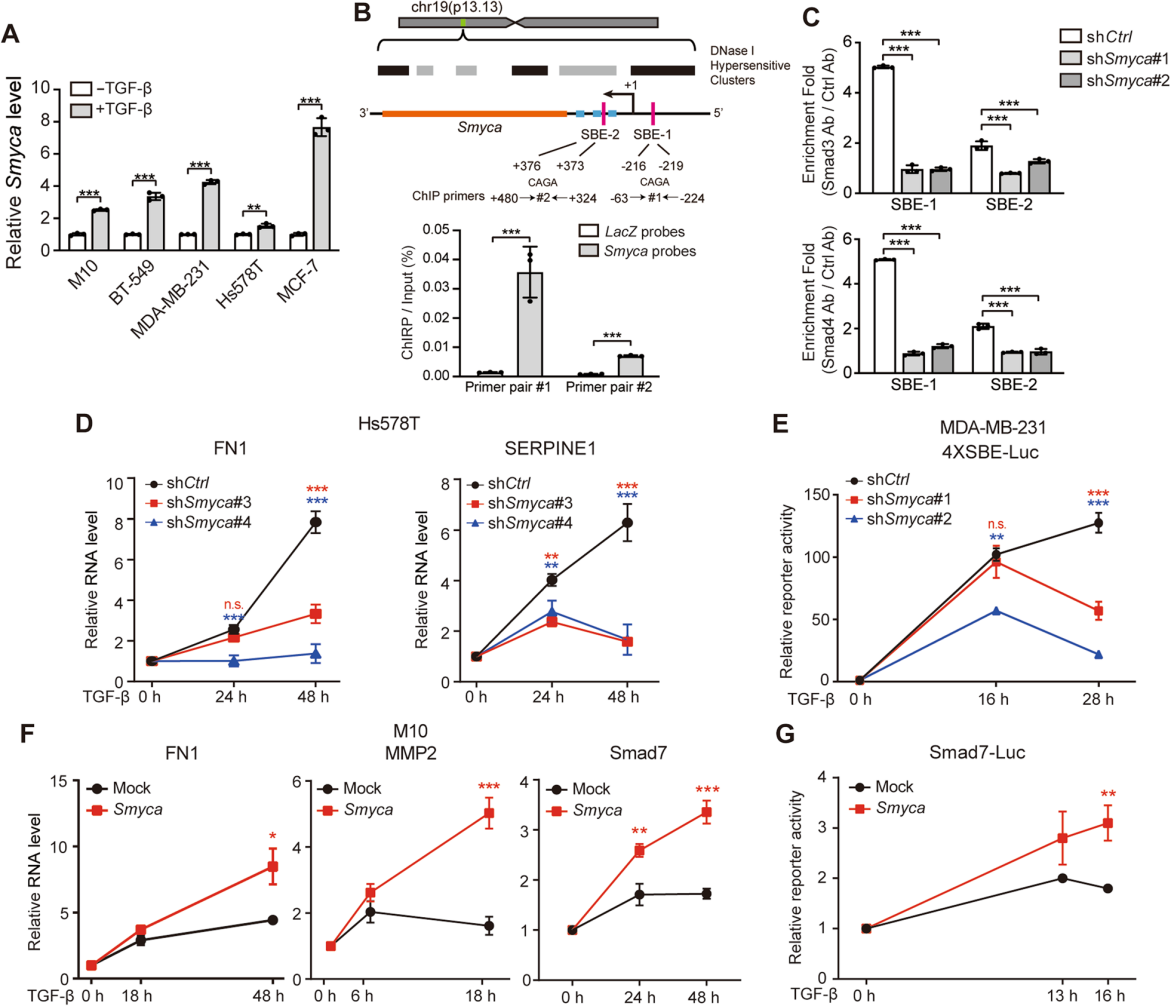
*Smyca* governs a positive feedback regulation of TGF-β signaling. (**A**) qRT-PCR analysis of *Smyca* expression in indicated cell lines treated with 5 ng/ml TGF-β for 6 h. Data are normalized with that of untreated group. (**B**) Upper panel: *Smyca* gene architecture. The transcription starting site (+ 1), regions corresponding to miR-23a ~ 27a ~ 24–2 clusters (blue), *Smyca* (orange) and locations of the two SBEs (red) and two sets of PCR primers covering the SBEs are indicated. The corresponding chromosome location of *Smyca* gene (green) and the DNase I hypersensitive clusters (gray and black) are shown on the top. Bottom panel: ChIRP assay for *Smyca* occupancy on its own promoter. Tilling biotinylated oligonucleotides complementary to *LacZ* or *Smyca* were used to pull down the RNA-associated chromatins from MDA-MB-231 cells treated with 5 ng/ml TGF-β for 2 h, followed by qRT-PCR analysis. Data are normalized with the inputs. The locations of two sets of PCR primers covering the two SBE regions of *Smyca* promoter are shown on the top. (**C**) ChIP analysis of the recruitment of Smad3 and Smad4 to the indicated SBEs in MDA-MB-231 cells stably expressing control or *Smyca* shRNAs and treated with 5 ng/ml TGF-β for 2 h. (**D**, **F**) qRT-PCR analysis of indicated genes in Hs578T cells stably expressing *Smyca* shRNAs (**D**) or M10 cells stably expressing *Smyca* (**F**) and treated with or without 5 ng/ml TGF-β for indicated time points. Data are normalized with that of untreated group (0 h). (**E**, **G**) Luciferase reporter assay on MDA-MB-231 cells stably expressing *Smyca* shRNAs (**E**) or M10 cells stably expressing *Smyca* (**G**) and treated with or without 5 ng/ml TGF-β for indicated time points. Data are normalized with that of untreated group (0 h). Data in all panels are mean ± SD, *n* = 3. *P* values are determined by unpaired *t* test (**A**, **B**, **F**, **G**) or one-way ANOVA with Tukey’s post hoc test (**C**, **D**, **E**), *P < 0.05, **P < 0.01, ***P < 0.001

Having identified an effect of *Smyca* on promoting Smad-mediated transcription, we next explored the conditions that regulate *Smyca* expression. Interestingly, *Smyca* expression was induced by TGF-β in multiple cell lines and Smad3 or Smad4 knockdown abolished TGF-β-induced *Smyca* expression (Fig. 4A and Additional file 1: Fig. S5A). Furthermore, two Smad-binding elements (SBEs) were found in 5’ regulatory and intra-gene regions of the *Smyca* gene. ChIRP and ChIP assays demonstrated the recruitment of *Smyca* and Smad3/Smad4 to the two regions, respectively (Fig. 4B, C). Furthermore, *Smyca* knockdown diminished the binding of Smad3 and Smad4 to these SBEs (Fig. 4C). These findings not only uncovered an autoregulatory mode of *Smyca* for its own transcription, but also suggested a role of *Smyca* in mediating a positive feedback regulation of TGF-β/Smad signaling. Accordingly, we showed that *Smyca* knockdown in Hs578T or MDA-MB-231 cells decreased the amplitude and duration of TGF-β-induced gene expression (Fig. 4D and Additional file 1: Fig. S5B). *Smyca* knockdown also shortened the responsive period for TGF-β-induced Smad-responsive reporter activity (Fig. 4E). Conversely, *Smyca* overexpression in M10 cells increased the amplitude and duration of TGF-β-induced gene expression and reporter activity (Fig. 4F, G). Thus, *Smyca* governs a positive feedback regulation of TGF-β/Smad signaling to enhance and prolong the signal output.

### *Smyca* activates c-Myc signaling by promoting chromatin recruitment of c-Myc/Max complex and c-Myc/TRRAP interaction

**Fig. 5:**
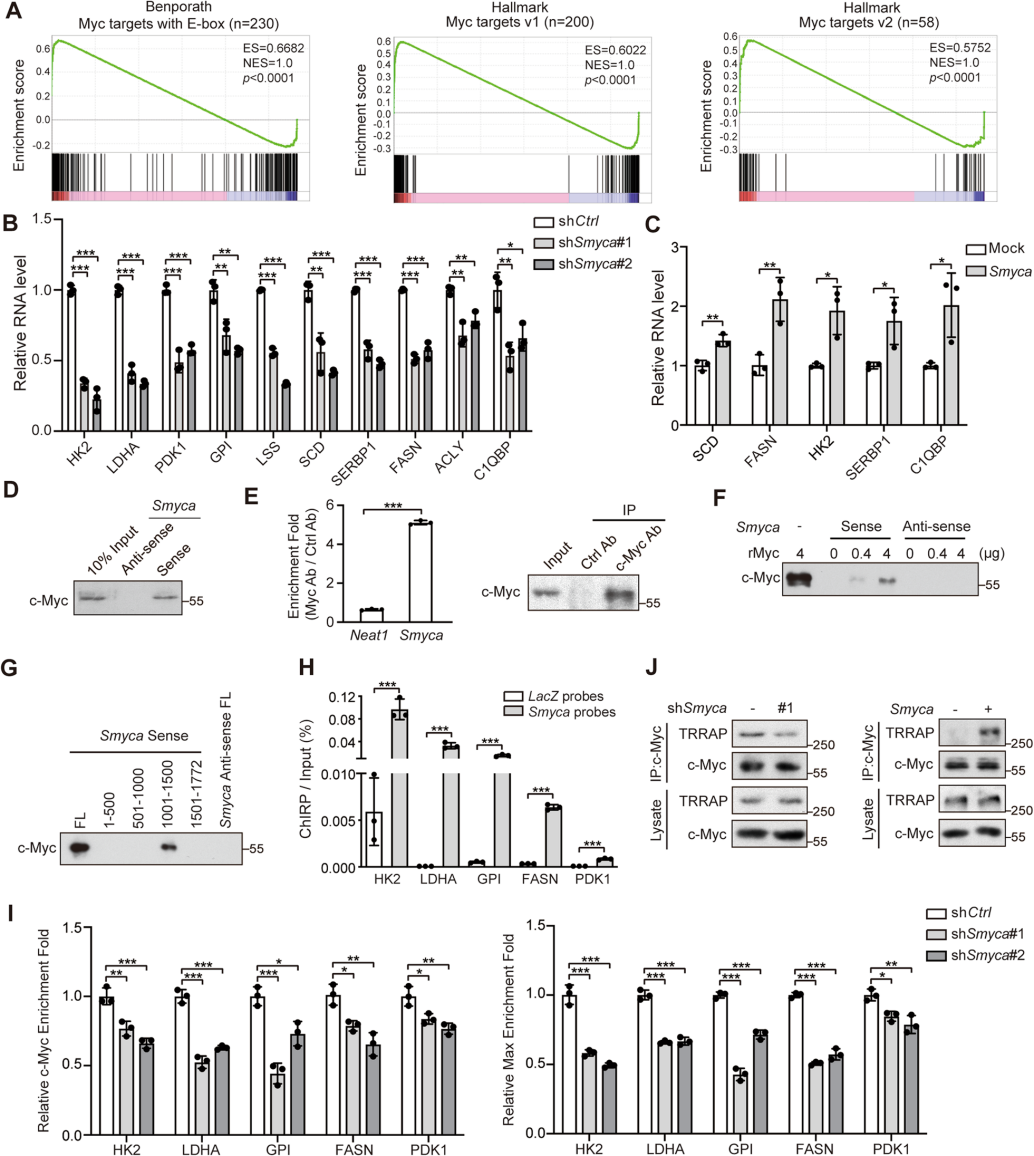
*Smyca* promotes c-Myc/Max complex recruitment to its target promoters and c-Myc/TRRAP binding. (**A**) GSEA plots for the match of *Smyca* signature with Myc signature from indicated sources. (**B**, **C**) qRT-PCR analysis of indicated c-Myc targets in MDA-MB-231 cells stably expressing *Smyca* shRNAs (**B**) or MCF7 cells stably expressing *Smyca* (**C**). Data are normalized with that derived from control cells. (**D**, **F**, **G**) RNA pull-down analysis by incubating MDA-MB-231 nuclear extracts (**D**, **G**) or indicated amounts of recombinant c-Myc protein (**F**) with biotinylated sense or antisense *Smyca* (**D**, **F**) or its deletion mutants (**G**). (**E**) RIP assay for the enrichment of *Smyca* in c-Myc immunoprecipitates derived from MDA-MB-231 cells. Data are normalized with that derived from *Neat1*. The presence of c-Myc in the immunoprecipitates is shown on the right panel. (**H**) ChIRP assay for detecting *Smyca* occupancy on indicated c-Myc target loci. Tilling biotinylated oligonucleotides complementary to *LacZ* or *Smyca* were used to pull down the RNA-associated chromatins from MDA-MB-231 cells, followed by qRT-PCR analysis. Data are normalized with that of inputs. (**I**) ChIP analysis of the recruitment of c-Myc or Max to indicated promoters in MDA-MB-231 cells stably expressing *Smyca* shRNAs. The enrichment folds are normalized with that from control cells. Data in (**B**), (**C**), (**E**), (**H**) and (**I**) are mean ± SD, *n* = 3. *P* values are determined by one-way ANOVA with Tukey’s post hoc test (**B**, **I**) or unpaired *t* test (**C**, **E**, **H**), *P < 0.05, **P < 0.01, ***P < 0.001. (**J**) Immunoprecipitation analysis of c-Myc binding to TRRAP in MDA-MB-231 cells stably expressing *Smyca* shRNA or *Smyca*

Besides TGF-β signaling, GSEA Hallmark Pathway analysis revealed that *Smyca*-regulated gene signature correlated significantly with Myc target signatures (Fig. 2D, blue). Furthermore, *Smyca-*regulated genes are enriched with genes in glycolysis and fatty acid metabolism (Fig. 2D, green), many of which are c-Myc targets [45]. In addition, *Smyca*-regulated gene signature correlated with the signature of E2F targets (Fig. 2D, green), which act downstream of c-Myc [46], as well as unfolded protein response (Fig. 2D, green), which is often induced by an unconstraint Myc activity in cancer cells [47]. Accordingly, GSEA analysis showed significant correlations of *Smyca*-induced gene signature with c-Myc target signature from multiple sources (Fig. 5A). These findings prompted us to investigate the impact of *Smyca* on c-Myc signaling. Importantly, *Smyca* knockdown in MDA-MB-231 or Malaru cells downregulated the expression of a set of c-Myc target genes (Fig. 5B and Additional file 1: Fig. S6A), most of which involve in cancer metabolic reprograming. In contrast, *Smyca* overexpression in MCF7 cells showed an opposite effect (Fig. 5C). Using a c-Myc-responsive luciferase reporter, we showed that *Smyca* overexpression increased the reporter activity, whereas *Smyca* knockdown diminished this activity (Additional file 1: Fig. S6B, C). Furthermore, by retrieving RNA-seq data from TCGA data sets, we found that *Smyca* expression levels positively correlated with the expression of a set of c-Myc target genes in the tumor tissues of breast cancer and HCC patients (Additional file 1: Fig. S6D), thus supporting the clinical relevance of *Smyca*-activated c-Myc signaling to human cancers. Together, our study identified a role of *Smyca* in promoting c-Myc signaling.

Next, we determined the mechanism underlying *Smyca*-promoted c-Myc signaling. RNA pull-down and RIP assays demonstrated a specific interaction of *Smyca* with c-Myc (Fig. 5D, E). Furthermore, purified recombinant c-Myc (rMyc) was readily pulled down by in vitro transcribed *Smyca* (Fig. 5F), indicating their direct interaction. We further showed that deletion of the transcriptional activating domain (TAD), but not DNA-binding domain, of c-Myc greatly reduced its binding to *Smyca*, whereas TAD alone was sufficient in binding *Smyca* (Additional file 1: Fig. S6E). Reciprocally, *Smyca* (1001-1500) region was responsible for binding c-Myc (Fig. 5G). Furthermore, deletion of this region abrogated the ability of *Smyca* to upregulate c-Myc-responsive reporter activity (Additional file 1: Fig. S6B). Thus, our study revealed a role of *Smyca* in binding c-Myc to enhance c-Myc-mediated gene expression. Notably, *Smyca* overexpression or knockdown affected neither the expression of c-Myc nor the ability of c-Myc to form a complex with Max (Additional file 1: Fig. S6F, G). Nevertheless, ChIRP assay demonstrated the occupancy of *Smyca* to a number of c-Myc target promoters (Fig. 5H). Furthermore, *Smyca* knockdown compromised the recruitment of both c-Myc and Max to these promoters (Fig. 5I). These findings suggest that *Smyca* guides c-Myc/Max complex to its target promoter. In addition to this effect, *Smyca* knockdown decreased c-Myc binding to TRRAP, which is required for c-Myc-mediated transcriptional activation by acting as a scaffold for bringing the STAGA and NuA4 histone acetyltransferase complexes [48]. In contrast, *Smyca* overexpression elevated c-Myc/TRRAP complex (Fig. 5J). Thus, our data identified dual roles of *Smyca* in activating c-Myc-mediated transcription, i.e., promoting the recruitment of c-Myc/Max complex to its target promoters and enhancing c-Myc binding to TRRAP.

### c-Myc and Smad3/4 form separate complexes with *Smyca* and compete for binding *Smyca*

**Fig. 6:**
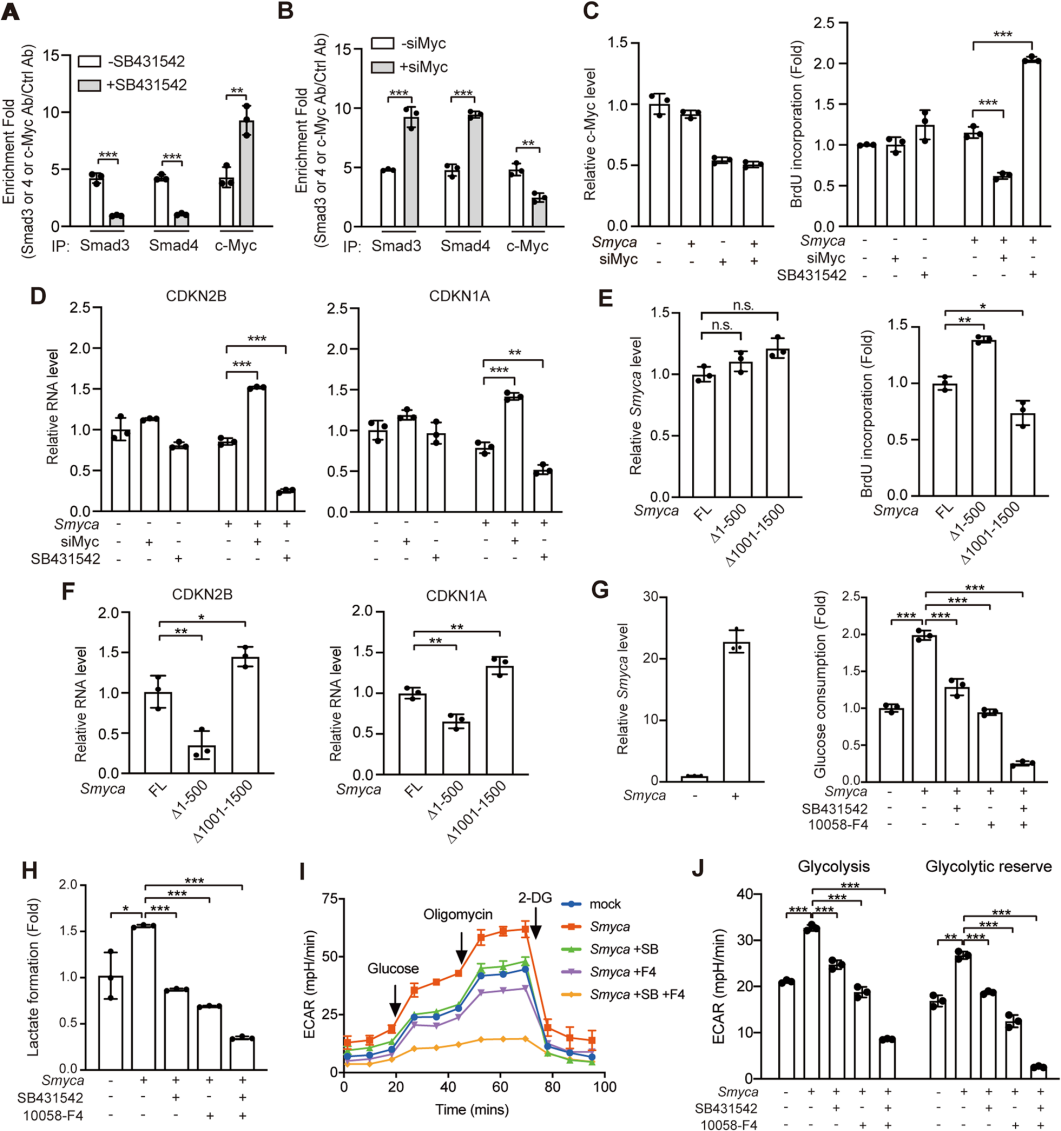
*Smyca* coordinates with TGF-β and c-Myc pathways for stimulating glycolysis and preventing growth inhibition. (**A**, **B**) RIP assays for the enrichment of *Smyca* in Smad3, Smad4 or c-Myc immunoprecipitates derived from MCF7 cells stably expressing *Smyca* and treated with or without 5 µM SB431542 for 24 h (**A**), or MCF7 cells stably expressing *Smyca* and transfected with c-Myc siRNA (**B**). (**C**, **D**) Cell proliferation (**C**) and qRT-PCR analysis (**D**) of MCF7 cells stably expressing *Smyca*, transfected with c-Myc siRNA, and/or treated with 5 µM SB431542 for 24 h. Validation of c-Myc knockdown efficiency is shown on the left panel in (**C**). (**E**, **F**) Cell proliferation (**E**) and qRT-PCR analysis (**F**) of MCF7 cells transfected with indicated *Smyca* constructs. The expression levels of *Smyca* and mutants are shown on the left panel in (**E**). (**G**, **H**) Glucose consumption (**G**) and lactate formation (**H**) in BT-549 cells transfected with *Smyca* and treated with 10 µM SB431542 and/or 150 µM 10058-F4 for 24 h (**G**) or 48 h (**H**). Validation of *Smyca* overexpression is shown on the left panel in (**G**). (**I**, **J**) Glycolysis stress profile (**I**) and glycolysis and glycolytic reserve rates (**J**) were measured using MDA-MB-231 cells transfected and treated as in (**G**). Data in all panels are mean ± SD, *n* = 3. *P* values are determined by unpaired *t* test (**A**, **B**), one-way (**C**, **D**, **E**, **F**) or two-way (**G**, **H**, **J**) ANOVA with Tukey’s post hoc test, *P < 0.05, **P < 0.01, ***P < 0.001; ns, not significant

A previous study reported the ability of c-Myc to form a complex with Smad3 [19]. Since *Smyca* uses different regions for binding Smad3/Smad4 and c-Myc, we determined whether *Smyca* could function as a bridge to enhance the interaction of c-Myc with Smad3. However, *Smyca* overexpression did not affect the interaction of c-Myc with Smad3 in TGF-β-stimulated cells (Additional file 1: Fig. S7A). Furthermore, while expression of *Smyca* (1-500) or *Smyca* (1001-1500) segment alone, i.e., the Smad-binding or c-Myc-binding region, induced the activities of Smad- or c-Myc-responsive reporters, respectively, combined expression of these two segments showed no additive effect (Additional file 1: Fig. S7B, C). This finding suggests that the two binding regions function independently. To further determine the role of *Smyca* in the interplay between Smad and c-Myc pathways, we investigated whether Smad3/4 and c-Myc compete for binding *Smyca*. Importantly, administration of TβRI inhibitor SB431542, which blocks Smad3 phosphorylation and Smad3/4 nuclear entry, increased *Smyca* binding to c-Myc, whereas c-Myc depletion by siRNA enhanced *Smyca* binding to Smad3/4 (Fig. 6A, B). Together, these findings suggest that Smad3/4 and c-Myc form separate complexes with *Smyca* to independently activate Smad and c-Myc signaling, respectively. However, Smad3/4 and c-Myc can compete for binding *Smyca* and thus blocking one axis shifts the balance to promote the other axis.

### ***Smyca*** coordinates c-Myc and TGF-β pathways to regulate tumor proliferation and glycolysis

The identification of *Smyca* as a coactivator for Smad- and c-Myc-mediated transcription is of a particular importance in cancer biology. Since the two pathways control cell proliferation in opposite directions, we propose that the *Smyca*/c-Myc axis would induce an effect to neutralize the growth inhibitory function of *Smyca*/Smad axis. Consistent with this hypothesis, while stable overexpression of *Smyca* in MCF7 cells only modestly affected proliferation, suppression of c-Myc expression by an siRNA or c-Myc activity by a chemical inhibitor (10058-F4) in *Smyca*-overexpressing MCF7 cells greatly decreased cell proliferation (Fig. 6C and Additional file 1: Fig. S7D). Conversely, blockage of TGF-β pathway by SB431542 stimulated proliferation of *Smyca*-overexpressing cells (Fig. 6C). Previous studies indicated that Smad and c-Myc are both recruited to the promoters of CDK inhibitors *CDKN2B* and *CDKN1A*. While Smad activates their transcription, c-Myc elicits a repressive effect [19–21, 49, 50]. Consistently, ChIP assay revealed that *Smyca* enhanced the recruitment of Smad3, Smad4 and c-Myc to the promoters of *CDKN2B* and *CDKN1A* and these effects were compromised by blocking TGF-β or c-Myc signaling with SB431542 or 10058-F4, respectively (Additional file 1: Fig. S7E, F). Furthermore, inhibiting or silencing c-Myc in *Smyca-*overexpressing MCF7 cells elevated the expression of these CDK inhibitors, whereas blocking TGF-β pathway showed opposite effects (Fig. 6D and Additional file 1: Fig. S7G). Besides breast cancer cells, blockage of c-Myc and TGF-β pathways in *Smyca*-overexpressing HCC cell line NTU-BL also showed contrasting effects on cell proliferation and the expression of cell cycle regulators *CDKN1A* and *CCNA1* (also known as cyclin A1) (Additional file 1: Fig. S7H, I). In line with the opposite roles of *Smyca*/Smad and *Smyca*/c-Myc axes in proliferation, the Smad-binding defective mutant *Smyca* (△1-500) induced a higher proliferation and lower expression of *CDKN2B* and *CDKN1A*, compared with full-length *Smyca,* whereas the c-Myc-binding defective mutant *Smyca* (△1001-1500) elicited opposite effects (Fig. 6E, F). Together, our data revealed that *Smyca* activates c-Myc signaling to avoid the growth inhibitory effect of *Smyca*/Smad axis.

Besides proliferation, c-Myc plays a key role in rewiring tumor cell metabolism and Warburg effect is among the best characterized metabolic alterations induced by c-Myc [45]. Notably, evidence has emerged that TGF-β pathway also promotes glycolysis [51]. Thus, we tested whether *Smyca* could act through TGF-β and c-Myc pathways to synergize glycolysis. *Smyca* overexpression in BT-549 cells greatly potentiated glucose consumption and lactate formation, the initial step and the final product of glycolysis, respectively, and these effects were attenuated by inhibiting either TGF-β or c-Myc pathway. Combined inhibition of both pathways further decreased glucose consumption and lactate formation (Fig. 6G, H). Furthermore, Seahorse analysis revealed a significant induction of ECARs (including glycolysis and glycolytic reserve) by *Smyca* overexpression. Again, blockage of TGF-β or c-Myc pathway alone diminished such effect, whereas blockage of both pathways showed a synergistic reduction effect (Fig. 6I, J). Thus, *Smyca* coactivates c-Myc and TGF-β pathways to synergize glycolysis.

### Targeting *Smyca* overcomes metastasis and chemoresistance

**Fig. 7:**
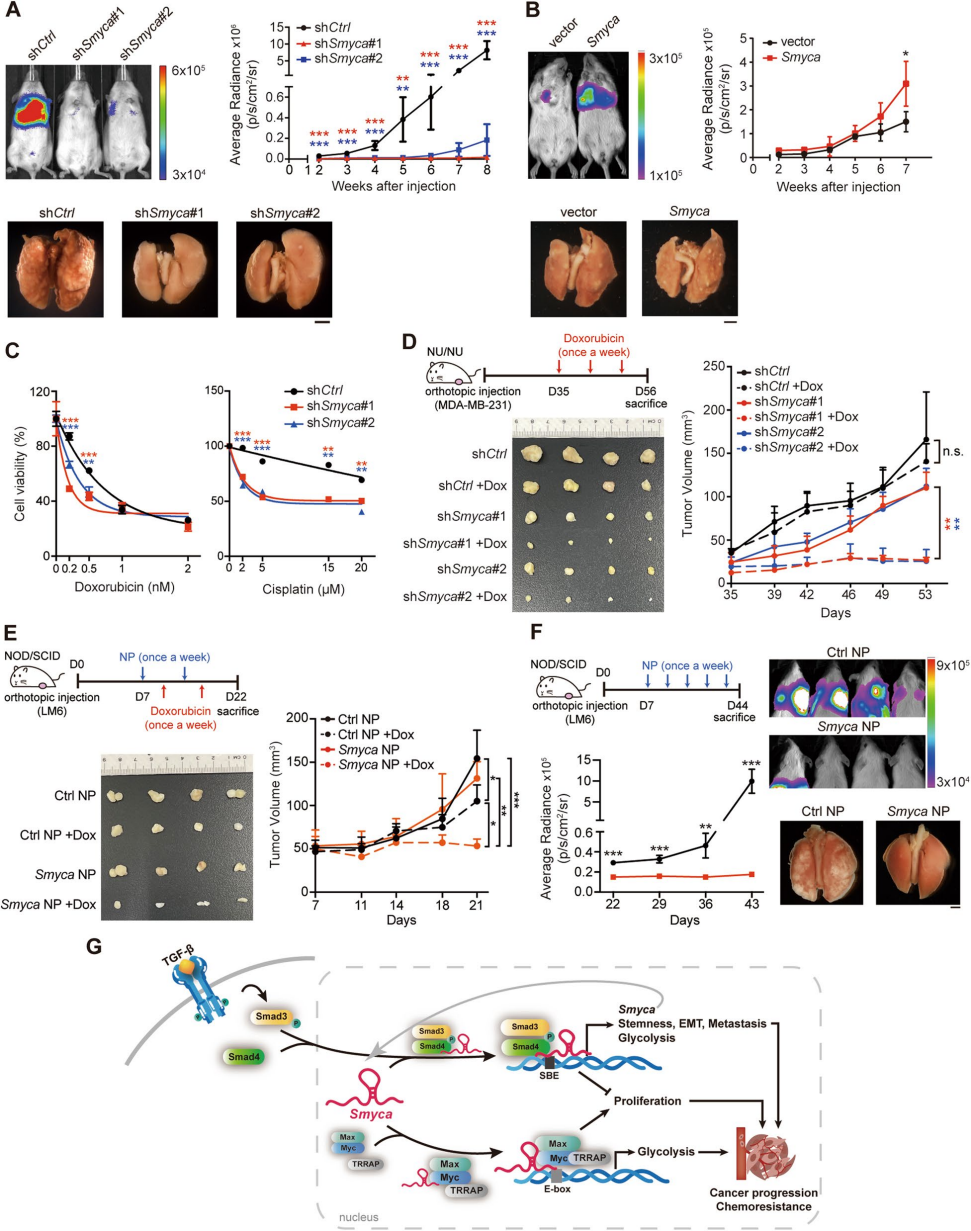
*Smyca* serves as a therapeutic target to prevent metastasis and chemoresistance. (**A**, **B**) Experimental metastasis assay for MDA-MB-231 cells stably expressing *Smyca* shRNAs (**A**) or *Smyca* (**B**). Representative images of the bioluminescence analysis at 8 weeks (**A**) or 7 weeks (**B**) after injection and the kinetics of metastasis at indicated time points are shown on the top left and right panels, respectively. The lung images at 8 weeks (**A**) and 7 weeks (**B**) after injection are shown on the bottom. Bar, 2 mm. (**C**) Cell viability assay of MDA-MB-231 cells stably expressing *Smyca* shRNAs and treated with Dox or cisplatin at indicated doses. (**D**) Nude mice orthotopically implanted with MDA-MB-231 cells stably expressing control or *Smyca* shRNAs and treated with Dox or DMSO as indicated (top left panel). Tumor volumes were measured at indicated days and plotted on the right. Tumors were surgically removed at the killing day and their sizes are shown on the bottom left panel. (**E**) NOD/SCID mice orthotopically implanted with LM6 cells were injected with NPs and/or Dox as indicated (top left panel). Tumor volumes at indicated days are shown on the right. Tumors were surgically removed at the killing day and their sizes are shown on the bottom left panel. (**F**) NOD/SCID mice orthotopically implanted with LM6 cells were injected with NPs as indicated (top left panel). Representative images of the bioluminescence analysis at 6 weeks after transplantation and the kinetics of metastasis at indicated time points are shown on the top right and bottom left panels, respectively. The lung images at 6 weeks after transplantation are shown on the bottom right. Bar, 2 mm. Data in (**A**), (**B**), (**C**), (**D**), (**E**), and (**F**) are mean ± SD, *n* = 5 (**A**), *n* = 4 (**B**, **D**, **E**, **F**), or *n* = 3 (**C**). *P* values are determined by one-way (**A**, **C**) or two-way (**D**, **E**) ANOVA with Tukey’s post hoc test or unpaired *t* test (**B**, **F**), *P < 0.05, **P < 0.01, ***P < 0.001; ns, not significant. (**G**) Schematic presentation of the roles of *Smyca* in coactivating TGF-β/Smad and Myc pathways to promote tumor progression

Our findings for the function of *Smyca* as a coactivator of TGF-β/Smad and c-Myc pathways strongly suggest its potential as a target for cancer therapy. Metastasis and drug resistance are the two leading causes of cancer mortality and both are associated with TGF-β-induced EMT [1, 2]. In addition, Warburg effect-induced acidification of tumor microenvironment potentiates metastasis [52]. We therefore tested the effects of *Smyca* on metastasis and chemoresistance. Using an experimental metastasis model by injecting tumor cells into the circulation of NOD/SCID mice, we found that *Smyca* knockdown in MDA-MB-231 cells greatly impaired lung metastasis (Fig. 7A). Conversely, *Smyca* overexpression in MDA-MB-231 cells enhanced metastasis (Fig. 7B). Next, we tested the effect of *Smyca* on chemoresistance. Using MTT assay, we showed that *Smyca* knockdown enhanced the sensitivity of MDA-MB-231 cells to Dox and cisplatin (Fig. 7C), two commonly used chemotherapeutic drugs in treating triple-negative breast cancer (TNBC) [53]. Similarly, in a breast cancer orthotopic model, Dox treatment almost completely blocked the growth of tumors derived from *Smyca* knockdown cells. However, tumor formed by parental cells showed little response to Dox (Fig. 7D). These findings collectively demonstrated the promoting effects of *Smyca* on metastasis and chemoresistance.

Next, we thought to develop a clinically applicable approach for targeting *Smyca*. To this end, we used *Smyca*-specific gapmer antisense oligonucleotides (ASO), which efficiently downregulated *Smyca* expression upon transfecting into MDA-MB-231 cells (Additional file 1: Fig. S8A). The *Smyca* gapmer ASO and control gapmer were loaded into LPD-NPs [40]. As to the TNBC model, we used LM6, a highly metastatic variant of MDA-MB-231 cells [32]. Incubation of LM6 cells with LPD-NPs carrying *Smyca* gapmer ASO dose-dependently reduced *Smyca* expression, compared with cells receiving control gapmer LPD-NPs (Additional file 1: Fig. S8B). Using an orthotopic model, we found that injection of *Smyca* gapmer ASO-loaded LPD-NPs into the NOD/SCID mice bearing LM6-derived tumors greatly enhanced the anti-tumor effect of Dox (Fig. 7E). Administration of *Smyca* gapmer ASO LPD-NPs in mice carrying orthotopically implanted LM6 cells reduced the size and weight of primary tumors and prevented lung metastasis (Fig. 7F and Additional file 1: Fig. S8C). qRT-PCR analysis of the primary tumors found that *Smyca* gapmer ASO LPD-NPs downregulated the expression of not only *Smyca*, but also a set of tumor-promoting TGF-β/Smad and c-Myc targets in vivo (Additional file 1: Fig. S8D). These findings highlight a potential of *Smyca* as an anticancer target and *Smyca* gapmer ASO as a promising agent for combating cancer metastasis and chemoresistance.

## Discussion

This study identifies *Smyca* as a lncRNA that coactivates two prominent pathways in controlling tumor malignancies, i.e., the TGF-β/Smad and c-Myc pathways, thereby driving tumor progression and therapy resistance. Mechanistically, the nuclear-residing *Smyca* functions as a scaffold to enhance the formation of Smad3/4 complex and its recruitment to a set of target promoters. Furthermore, *Smyca* binds c-Myc to promote the association of c-Myc with TRRAP and the recruitment of c-Myc/Max complex to some of target promoters. Through these mechanisms, *Smyca* not only potentiates multiple cancer hallmarks induced by the two pathways, including metabolic reprogramming, migration, invasion, cancer stemness, metastasis and chemoresistance, but also evades TGF-β-induced growth inhibition by stimulating the c-Myc proliferating signal (Fig. 7G). Consistent with these tumor-promoting effects, *Smyca* high expression correlates with poor prognosis and aggressiveness of multiple cancer types. Furthermore, targeting *Smyca* by the gapmer ASO approach efficiently blocks tumor metastasis and sensitizes tumor cells to chemotherapy. These findings highlight the prognostic and therapeutic values of *Smyca* in human cancers.

*Smyca* not only promotes TGF-β/Smad signaling but is itself a transcriptional target of Smad (Fig. 7G). This mutual reinforcement establishes a positive feedback loop to elevate the amplitude and duration of TGF-β signaling. Of note, a previous study identified the differential cellular responses to TGF-β treatment at early and late time points. At the late phase, R-Smad is redirected to the promoters of a set of invasion genes via a cooperation with JUNB [43], thus suggesting a role of sustained TGF-β signaling in favoring a tumor-promoting outcome. Similarly, HCC patients with a late TGF-β signature are associated with invasive phenotype, metastasis and poor prognosis [54]. Consistent with these previous studies, *Smyca* enhances TGF-β-induced expression of many invasion genes and *Smyca* expression correlates positively with the expression of a large set of invasion/metastasis genes in cancer patients. Thus, the ability of *Smyca* to govern a positive feedback regulation of TGF-β signaling may contribute in part to its ability to switch the dichotomous functions of TGF-β toward tumor promotion.

Intriguingly, *Smyca* binds Smad3 but not Smad2, which is in analogous to another lncRNA, *ELIT-1* [55]. Notably, a number of studies have reported the distinct roles of these two Smads in cancer progression. While Smad3 elicits pro-tumor functions by enhancing EMT, invasion and angiogenesis, Smad2 displays opposite effects in many scenarios to suppress tumor progression [8, 56–58]. These differential roles of the two Smads may be attributed to the differences in the affinity to promoters of target genes or in the recruitment of partners or regulators [58, 59]. Our finding that *Smyca* binds Smad3 but not Smad2 to elevate the expression of pro-tumor effectors of Smad not only is consistent with these previous studies but also suggests *Smyca* as one factor that contributes to the differential effects of Smad2 and Smad3 on tumor progression. Through the ability to govern a positive feedback regulation of TGF-β signaling to prolong the signal duration, *Smyca* would endow its binding partner Smad3 with a preference to turn on the expression of a set of invasion genes. In this way, Smad3 could outcompete Smad2 for preferentially gaining a tumor-promoting function.

Consistent with its distribution to the chromatins [28], *Smyca* not only is recruited to the promoters of a set of Smad3/4 and c-Myc targets, but enhances the recruitment of Smad3/4 and c-Myc/Max complexes to these loci. Since *Smyca* also potentiates Smad3/4 complex formation, we cannot exclude the possibility that the latter is merely a consequence of the former. However, due to the lack of effect of *Smyca* on c-Myc/Max association, our findings do support a role of *Smyca* in guiding the c-Myc/Max complex to some of their target promoters. It remains unclear whether *Smyca* is recruited to c-Myc and Smad3/4 target loci directly or indirectly via other factors, such as modified histones or chromatin remodeling factors. Intriguingly, *Smyca* also enhances c-Myc binding to TRRAP, which recruits histone acetyltransferase complexes to promote H3/4 acetylation and transcription [48]. Future studies are needed to investigate the order and interdependency for the loading of *Smyca*, c-Myc/Max and TRRAP to c-Myc target promoters as well as the choreography of the *Smyca*/c-Myc/TRRAP complex.

*Smyca* binds Smad3/4 and c-Myc via non-overlapping regions, and each region is sufficient and necessary for activating Smad- or c-Myc-mediated transcription. Furthermore, the two regions act independently and no additive effect is observed. Accordingly, *Smyca* does not promote Smad3/4 interaction with c-Myc, suggesting that *Smyca* forms separate complexes with Smad3/4 and c-Myc. Nevertheless, depleting c-Myc results in an elevation of *Smyca*/Smad3/4 complex, while depleting nuclear Smad3/4 by TβRI inhibitor increases *Smyca*/c-Myc complex. These findings reveal the existence of a competition between Smad3/4 and c-Myc for binding *Smyca*. Such competition might be resulted from the following scenarios. First, *Smyca* may be a limiting factor in cells in relation to Smad3/4 and c-Myc. Therefore, when *Smyca* binds to one partner and is recruited to the target promoters of this partner, it would limit the chance of *Smyca* to be recruited to the target promoters of another partner. Additionally, although Smad3/4 and c-Myc bind to separate regions in *Smyca*, the binding of one partner may recruit additional factors and/or alter the conformation of *Smyca*, thereby masking or eliminating the binding site of another partner. Regardless of the underlying mechanisms, this competitive binding is consistent with our finding that blockage of one pathway readily shifts the effects of *Smyca* on cell proliferation and the expression of cell cycle regulators. Thus, *Smyca* forms separate complexes with Smad3/4 and c-Myc and their competitive binding could determine the effect of *Smyca* on cell proliferation.

It is unclear whether *Smyca* acts on all target loci of Smad3/Smad4 and c-Myc/Max complexes or particular subsets of them. Intersection of *Smyca*-induced DEGs with published Smad2/3 ChIP-seq data revealed partial overlaps, suggesting the latter possibility. However, one caveat is that the DEGs and ChIP-seq data were derived from different cell types and treatment conditions. It would require further omics studies, such as the comparative ChIRP-seq and ChIP-seq analyses, conducted on matched cellular contexts to address this important issue. Lastly, although p-Smad3 readily binds Smad4 via their MH2 domains, *Smyca* interacts with the MH1 domains of the two Smads to further enhance their interaction and thus functions as a RNA scaffold. Even though the Smad3- and Smad4-binding regions are both located to the *Smyca* (1–500) segment, we postulate that non-overlapping regions in this segment are responsible for binding Smad3 and Smad4, thereby fulfilling a scaffold function. Notably, the ability of *Smyca* to enhance the association of a protein complex by providing an additional binding surface is in analogous to another lncRNA *GUARDIN*, which binds both BRCA1 and BARD1 to potentiate their association [60].

LncRNAs have emerged as a new class of biomarkers for cancer diagnosis and prognosis [23]. We uncover a remarkable value of *Smyca* for the prognosis of a number of cancer types. Notably, a recent study reported an upregulation of *Smyca* level in the patient plasma of several cancer types [31], implying the utility of *Smyca* as a noninvasive diagnostic biomarker. Besides diagnosis and prognosis, lncRNAs are also great targets of cancer therapy, because drugs targeting RNAs are easier design and synthesis than drugs targeting proteins. The ability of *Smyca* to activate two cancer-relevant pathways for potentiating multiple cancer hallmarks highlights its potential as a target for cancer therapy. In support of this notion, we show that NP-mediated delivery of *Smyca* gapmer ASO to the tumor-bearing mice significantly blocks tumor metastasis and overcomes chemoresistance, two major causes of cancer mortality.

## Conclusions

In conclusion, our study identifies *Smyca* as a lncRNA that coactivates TGF-β/Smad and c-Myc pathway to potentiate tumor progression, metastasis and chemoresistance. Furthermore, our findings reveal a great promise for the clinical applications of *Smyca* as a prognostic biomarker and a therapeutic target for certain cancer types.

## Supplementary Information

**Additional file 1. Figure S1.** *Smyca* expression correlates with poor prognosis. (**A**-**F**) Kaplan-Meier analysis of *Smyca* expression in relation to the overall survival (**A**, **C**, **E**, **F**) and disease-free survival (**B**, **D**) of indicated cancer types. Data were retrieved from TCGA data sets (**A**, **B**) or GEO data sets (**C**, **D**, **E**), or generated from an in house cohort (**F**). Patients were grouped into high and low expression based on the median expression level. Patients without survival information were omitted. Hazard ratio (HR) and *P* values are determined by log-rank test. (**G**) *Smyca* expression in HCC tumor tissues and adjacent normal tissues analyzed by qRT-PCR. (**H**–**K**) The correlation of *Smyca* expression with the stage (**H**, **J**) and invasiveness (**I**, **K**) of indicated cancer types. Data in (**G**-**K**) are presented by Whiskers boxplot, whiskers: min to max, bound of box: lower and upper quartiles, center line: median. Data points derived from basal-like subgroup of patients are marked in blue in (**K**). *P* values in (**G**), (**H**), (**I**), (**J**), (**K**) are determined by unpaired t-test. (**L**) *Smyca* expression in different subtypes of breast cancer patients analyzed by qRT-PCR. Data are expressed as mean ± SD. *P* values are determined by oneway ANOVA with Tukey’s post hoc test. **Figure S2.** *Smyca* promotes EMT. (**A**) *Smyca* expression in different breast cancer and HCC cell lines analyzed by qRT-PCR and represented as copy numbers. Data are expressed as mean ± SD from three independent experiments. (**B**) qRT-PCR analysis of indicated miRNAs in MDA-MB-231 cells stably expressing various *Smyca* shRNAs. Data are normalized with that of control cells and are expressed as mean ± SD from three independent experiments, ns, not significant by one-way ANOVA with Tukey’s post hoc test. (**C**, **D**) Western blot analysis of EMT markers in Hs578T cells stably expressing *Smyca* shRNAs (**C**) or MCF7 cells stably overexpressing *Smyca* (**D**). The amounts of each protein in relation to the control cells are indicated under the bands. *Smyca* expression levels in these stable lines are shown on the left panels. Data are mean ± SD from three independent experiments. (**E**-**H**) Cell proliferation (**E**, **F**) and cell viability (**G**, **H**) assays of M10 cells stably expressing *Smyca* (**E**, **G**) or MDA-MB-231 cells stably expressing *Smyca* shRNAs (**F**, **H**). Data are mean ± SD, n=3. *P* values are determined by unpaired t-test (**E**, **G**) or one-way ANOVA with Tukey’s post hoc test (**F**, **H**), ns, not significant. **Figure S3.** Bioinformatics and cell-based analysis for the relation of *Smyca* to TGF-β signaling. (**A**) Summary of the GSEA analysis for the match of *Smyca* signature with the indicated signatures. Data origin indicates the source database or the first author identifying the gene signature. (**B**) Intersection of *Smyca*-induced DEGs derived from MDA-MB-231 cells with Smad2/3 ChIP-seq data derived from Hs578T cells and BT-549 cells. The full list of overlapped genes is shown in Table S5. (**C**) qRT-PCR analysis for the expression of indicated TGF-β target genes in Hs578T cells stably expressing *Smyca* shRNAs and treated with or without 5 ng/ml TGF-β for 48 hr. Data are normalized with that of untreated group in each cell. (**D**, **E**) Luciferase assay for the Smad-target reporters transfected into Hs578T cells (**D**) or Malaru cells (**E**) stably expressing *Smyca* shRNAs and treated with or without 5 ng/ml TGF-β for 24 hr. The *Smyca* knockdown efficiencies are shown on the left panel in (**E**). Data in (**C**), (**D**), (**D**) are normalized with that of untreated control and presented as mean ± SD, n=3. *P* values are determined by one-way ANOVA with Tukey’s post hoc test, **P < 0.01, ***P < 0.001. (**F**, **G**) Summary of the correlations of *Smyca* expression with the expression of indicated TGF-β target genes by analyzing HCC or breast cancer data sets from TCGA (**F**) or GEO (**G**) databases. Pearson’s coefficients and *P* values are indicated. (**H**) GO analysis using the set of TGF-β target genes with expression levels showing positive correlations with *Smyca* expression in HCC and/or breast cancers. **Figure S4.** *Smyca* binds MH1 domains of Smad3 and Smad4 without affecting their expression and Smad3 phosphorylation. (**A**) Western blot analysis of Smad3 and Smad4 expression in MDA-MB-231 cells stably expressing *Smyca* shRNAs. (**B**) Western blot analysis for Smad3 phosphorylation in MDA-MB-231 cells stably expressing *Smyca* shRNAs and treated with or without 5 ng/ml TGF-β for 30 min. The blots are representative of three independent experiments and quantitative data are shown on the right. (**C**) Immunoprecipitation analysis of Smad3 and Smad4 interaction in MDA-MB-231 cells stably expressing indicated *Smyca* constructs and treated with or without 5 ng/ml TGF-β for 1 hr. (**D**) Luciferase assay for the Smad-responsive reporter in MDA-MB-231 cells transfected with indicated *Smyca* constructs and treated with or without 5 ng/ml TGF-β for 24 hr. The expression levels of *Smyca* are shown on the left panel. (**E**) Baculovirally purified Smad3, Smad4, and their MH1 deletion mutants bound on beads were incubated with biotinylated sense or antisense *Smyca*. The bound *Smyca* was analyzed by qRT-PCR. The equal inputs of recombinant proteins are shown on or antisense *Smyca*. The bound *Smyca* was analyzed by qRT-PCR. The equal inputs of recombinant proteins are shown on the right. Data in (**B**), (**D**) and (**E**) are mean ± SD, n=3. *P*values are determined by one-way ANOVA with Tukey’s post hoc test (**B**, **D**) or unpaired t-test (**E**), *** *P* < 0.001; ns., not significant. **Figure S5.** *Smyca* is a Smad target and mediates a positive feedback control of TGF-β signaling. (**A**) qRT-PCR analysis of *Smyca* expression in MDA-MB-231 cells stably expressing Smad3 or Smad4 shRNAs and treated with or without 5 ng/ml TGF-β for 24 hr. The knockdown efficiencies of Smad3 and Smad4 shRNAs are shown on the left and middle panels, respectively. (**B**) qRT-PCR analysis of TGF-β target gene expression in MDA-MB-231 cells stably expressing *Smyca* shRNAs and treated with or without 5 ng/ml TGF-β for indicated time points. Data are normalized with that of untreated group (0 h). Data in (**A**) and (**B**) are mean ± SD, n=3. *P* values are determined by unpaired t-test (**A**) or one-way ANOVA with Tukey’s post hoc test (**B**), *** *P* < 0.001. **Figure S6.** *Smyca* promotes c-Myc transcription activity without affecting its expression or interaction with Max. (**A**) qRT-PCR analysis of the expression of indicated c-Myc target genes in Malaru cells stably expressing control or *Smyca* shRNA. Data are normalized with that of control cells. (**B**, **C**) Luciferase assay for a c-Myc-reponsive reporter transfected into MDA-MB-231 cells stably expressing *Smyca* or mutant (**B**) or *Smyca* shRNAs (**C**). The expression levels of *Smyca* and its mutant are shown on the left panel in (**B**). (**D**) Summary of the correlations of *Smyca* expression with the expression of indicated c-Myc target genes by analyzing breast cancer and HCC data sets from TCGA. Pearson’s coefficients and *P* values are indicated. (**E**) Maping the *Smyca*-binding region in c-Myc. Top: Schematic presentaton of c-Myc domains. The various GFP-c-Myc truncated proteins were purified from transfected 293T cells and incubated with biotinylated sense or antisense *Smyca*. The bound *Smyca* was analyzed by qRT-PCR. Data are normalized with that from GFP only control. The input levels of various GFP fusion proteins are shown on the right and marked by arrows. (**F**) Western blot analysis of c-Myc expression in indicated cells stably expressing *Smyca* or *Smyca* shRNAs. (**G**) Immunoprecipitation analysis of c-Myc/Max complex formation in MDA-MB-231 cells stably expressing *Smyca* or *Smyca* shRNA. Data in (**A**), (**B**), (**C**), (**E**) are mean ± SD, n=3. *P* values are determined by unpaired t-test (**A**), or one-way ANOVA with Tukey’s post hoc test (**B**, **C**, **E**), *P < 0.05, **P < 0.01, *** *P* < 0.001. **Figure S7.** *Smyca*-promoted c-Myc singaling neutralizes the growth inhibitory effect of *Smyca*-promoted TGF-β signaling. (**A**) Immunoprecipitation analysis of c-Myc/Smad complex formation in MDA-MB-231 cells stably expressing *Smyca* and treated with 5 ng/ml TGF-β for 2 hr. (**B**, **C**) Luciferase assay for a Smad-responsive reporter (**B**) or c-Myc-responsive reporter (**C**) transfected into MDA-MB-231 cells together with indicated *Smyca* constructs and treated with or without 5 ng/ml TGF-β for 24 hr. The expression levels of *Smyca* (1-500) and (1001-1500) fragments are shown on the left and middle panels in (**B**), respectively. (D, H) Cell proliferation assay of MCF7 cells stably expressing *Smyca* and treated with 75 μM 10058-F4 for 24 hr (**D**) or NTU-BL cells stably expressing *Smyca* and treated with 5 μM SB431542 or 75 μM 10058-F4 for 24 hr (**H**). (**E**, **F**) ChIP analysis for Smad3, Smad4, and c-Myc binding to the promoter regions of CDKN2B (E) and CDKN1A (F) genes in MDA-MB-231 cells stably expressing *Smyca* and treated with 5 μM SB431542 and/or 150 μM 10058-F4 for 2 hr. (G, **I**) qRTPCR analysis of the expression of indicated genes in MCF7 (**G**) or NTU-BL (**I**) cells stably expressing *Smyca* and treated with 5 μM SB431542 or 75 μM 10058-F4 for 24 hr. *Smyca* expression levels are shown on the left panel of (**I**). Data in (**B**), (**C**), (**D**), (**E**), (**F**), (**G**), (**H**), (**I**) are mean ± SD, n=3. *P* values are determined by one-way ANOVA with Tukey’s post hoc test, *P < 0.05, **P < 0.01, ***P < 0.001. **Figure S8.** Downregulation of the expression of *Smyca*, Smad targets, and c-Myc targets by *Smyca* gapmer ASO. (**A**, **B**, **D**) qRT-PCR analysis of *Smyca* or indicated mRNAs in MDA-MB-231 cells transfected with *Smyca* gapmer ASO (**A**), LM6 cells treated with indicated doses of NPs carrying *Smyca* gapmer ASO or control gapmer (**B**), or LM6 tumor-bearing mice treated with NPs carrying *Smyca* gapmer ASO or control NPs (**D**). (**C**) The morphology, size and weight of primary tumors taken from the sacrifice day for experiment shown in Fig. 7F. Data in all panels are mean ± SD, n=3 (**A**, **B**, **D**) or 4 (**C**). *P* values are determined by unpaired t-test, *P < 0.05, **P < 0.01, ***P < 0.001. **Table S1**. Antibody details. **Table S2**. siRNA, shRNA and Gapmer sequences. **Table S3**. Sequences of PCR primers. **Table S4**. ChIRP probe sequences. **Table S5**. List of genes that are regulated by *Smyca* and bound by Smad2/3.

## Data Availability

All data needed to evaluate the conclusions in the paper are present in the paper and/or Additional file 1. The RNA-seq data were deposited to the GEO database with the accession number GSE181028. Other data and materials are available upon reasonable request.

## Abbreviations

ASO: Antisense oligonucleotides
CCNA1: Cyclin A1
ChIP: Chromatin immunoprecipitation
ChIRP: Chromatin isolation by RNA purification
DEGs: Differentially expressed genes
DMEM: Dulbecco’s modified Eagle’s medium
Dox: Doxorubicin
ECAR: Extracellular acidification rate
ELDA: Extreme limiting dilution assay
EMT: Epithelial–mesenchymal transition
FBS: Fetal bovine serum
FDR: False discovery rate
FN1: Fibronectin1
GEO: Gene expression omnibus
GSEA: Gene set enrichment analysis
HCC: Hepatocellular carcinoma
IPA: Ingenuity pathway analysis
LncRNA: Long noncoding RNA
LPD-NPs: Liposome–polycation–DNA nanoparticles
MET: Mesenchymal-to-epithelial transition
MTT: Methyl thiazolyl diphenyl tetrazolium bromide
PMSF: Phenylmethylsulphonyl fluoride
RIP: RNA immunoprecipitation
rMyc: Recombinant c-Myc
RNA-seq: RNA-sequencing
qRT-PCR: Real-time quantitative PCR
qPCR: Quantitative PCR
SBE: Smad-binding elements
*Smyca*: Smad/Myc coactivator
TAD: Transcriptional activating domain
TβRI: Type I TGF-β receptor
TCGA: The cancer genome atlas
TNBC: Triple-negative breast cancer
