# Supplementary material for: Long noncoding RNA Smyca coactivates TGF-β/Smad and Myc pathways to drive tumor progression
Source: J Hematol Oncol. 2022 Jul 6;15:85. doi: 10.1186/s13045-022-01306-3 (PMC9258208; doi:10.1186/s13045-022-01306-3)
Supplement: Supplementary file 1 — Additional file 1. Figure S1. Smyca expression correlates with poor prognosis. (A-F) Kaplan-Meier analysis of Smyca expression in relation to the overall survival (A, C, E, F) and disease-free survival (B, D) of indicated cancer types. Data were retrieved from TCGA data sets (A, B) or GEO data sets (C, D, E), or generated from an in house cohort (F). Patients were grouped into high and low expression based on the median expression level. Patients without survival information were omitted. Hazard ratio (HR) and P values are determined by log-rank test. (G) Smyca expression in HCC tumor tissues and adjacent normal tissues analyzed by qRT-PCR. (H–K) The correlation of Smyca expression with the stage (H, J) and invasiveness (I, K) of indicated cancer types. Data in (G-K) are presented by Whiskers boxplot, whiskers: min to max, bound of box: lower and upper quartiles, center line: median. Data points derived from basal-like subgroup of patients are marked in blue in (K). P values in (G), (H), (I), (J), (K) are determined by unpaired t-test. (L) Smyca expression in different subtypes of breast cancer patients analyzed by qRT-PCR. Data are expressed as mean ± SD. P values are determined by oneway ANOVA with Tukey’s post hoc test. Figure S2. Smyca promotes EMT. (A) Smyca expression in different breast cancer and HCC cell lines analyzed by qRT-PCR and represented as copy numbers. Data are expressed as mean ± SD from three independent experiments. (B) qRT-PCR analysis of indicated miRNAs in MDA-MB-231 cells stably expressing various Smyca shRNAs. Data are normalized with that of control cells and are expressed as mean ± SD from three independent experiments, ns, not significant by one-way ANOVA with Tukey’s post hoc test. (C, D) Western blot analysis of EMT markers in Hs578T cells stably expressing Smyca shRNAs (C) or MCF7 cells stably overexpressing Smyca (D). The amounts of each protein in relation to the control cells are indicated under the bands. Smyca expression levels [file 13045_2022_1306_MOESM1_ESM.docx]

**Additional file 1: Figure Legends and Tables**

**Figure S1. *Smyca* expression correlates with poor prognosis.** (**A-F**) Kaplan-Meier analysis of *Smyca* expression in relation to the overall survival (**A, C, E, F**) and disease-free survival (**B, D**) of indicated cancer types. Data were retrieved from TCGA data sets (**A, B**) or GEO data sets (**C, D, E**), or generated from an in house cohort (**F**). Patients were grouped into high and low expression based on the median expression level. Patients without survival information were omitted. Hazard ratio (HR) and *P* values are determined by log-rank test. **(G)** *Smyca* expression in HCC tumor tissues and adjacent normal tissues analyzed by qRT-PCR. (**H-K**) The correlation of *Smyca* expression with the stage (**H, J**) and invasiveness (**I, K**) of indicated cancer types. Data in (**G-K**) are presented by Whiskers boxplot, whiskers: min to max, bound of box: lower and upper quartiles, center line: median. Data points derived from basal-like subgroup of patients are marked in blue in (**K**). *P* values in (**G**), (**H**), (**I**), (**J**), (**K**) are determined by unpaired t-test. (**L**) S*myca* expression in different subtypes of breast cancer patients analyzed by qRT-PCR. Data are expressed as mean ± SD. *P* values are determined by one-way ANOVA with Tukey’s post hoc test.


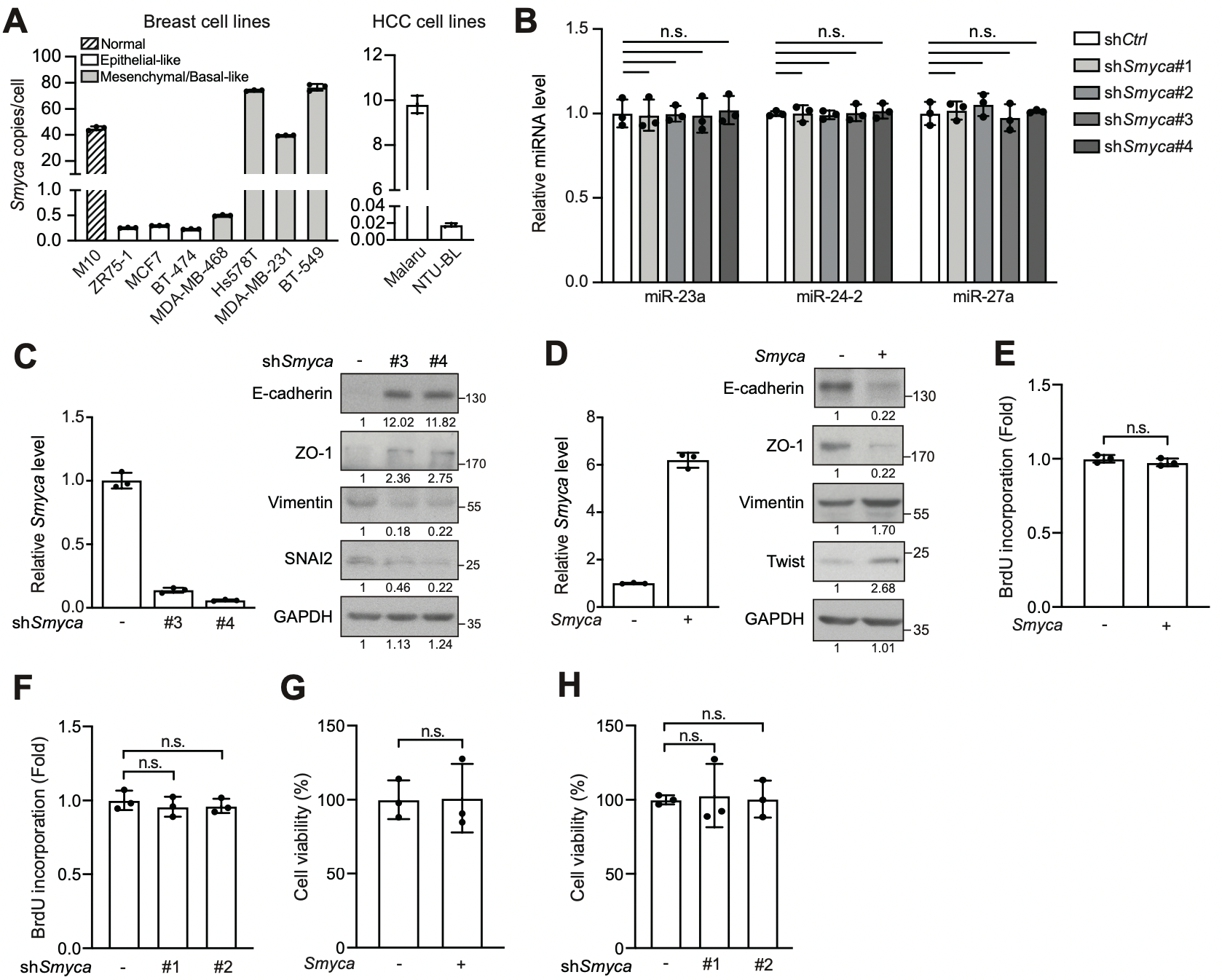


**Figure S2. *Smyca* promotes EMT.** **(A)** *Smyca* expression in different breast cancer and HCC cell lines analyzed by qRT-PCR and represented as copy numbers. Data are expressed as mean ± SD from three independent experiments. **(B)** qRT-PCR analysis of indicated miRNAs in MDA-MB-231 cells stably expressing various *Smyca* shRNAs. Data are normalized with that of control cells and are expressed as mean ± SD from three independent experiments, ns, not significant by one-way ANOVA with Tukey’s post hoc test. **(C, D)** Western blot analysis of EMT markers in Hs578T cells stably expressing *Smyca* shRNAs **(C)** or MCF7 cells stably overexpressing *Smyca* **(D)**. The amounts of each protein in relation to the control cells are indicated under the bands. *Smyca* expression levels in these stable lines are shown on the left panels. Data are mean ± SD from three independent experiments. (**E-H**) Cell proliferation (**E, F**) and cell viability (**G, H**) assays of M10 cells stably expressing *Smyca* (**E, G**) or MDA-MB-231 cells stably expressing *Smyca* shRNAs (**F, H**). Data are mean ± SD, n=3. *P* values are determined by unpaired t-test (**E, G**) or one-way ANOVA with Tukey’s post hoc test (**F, H**), ns, not significant.


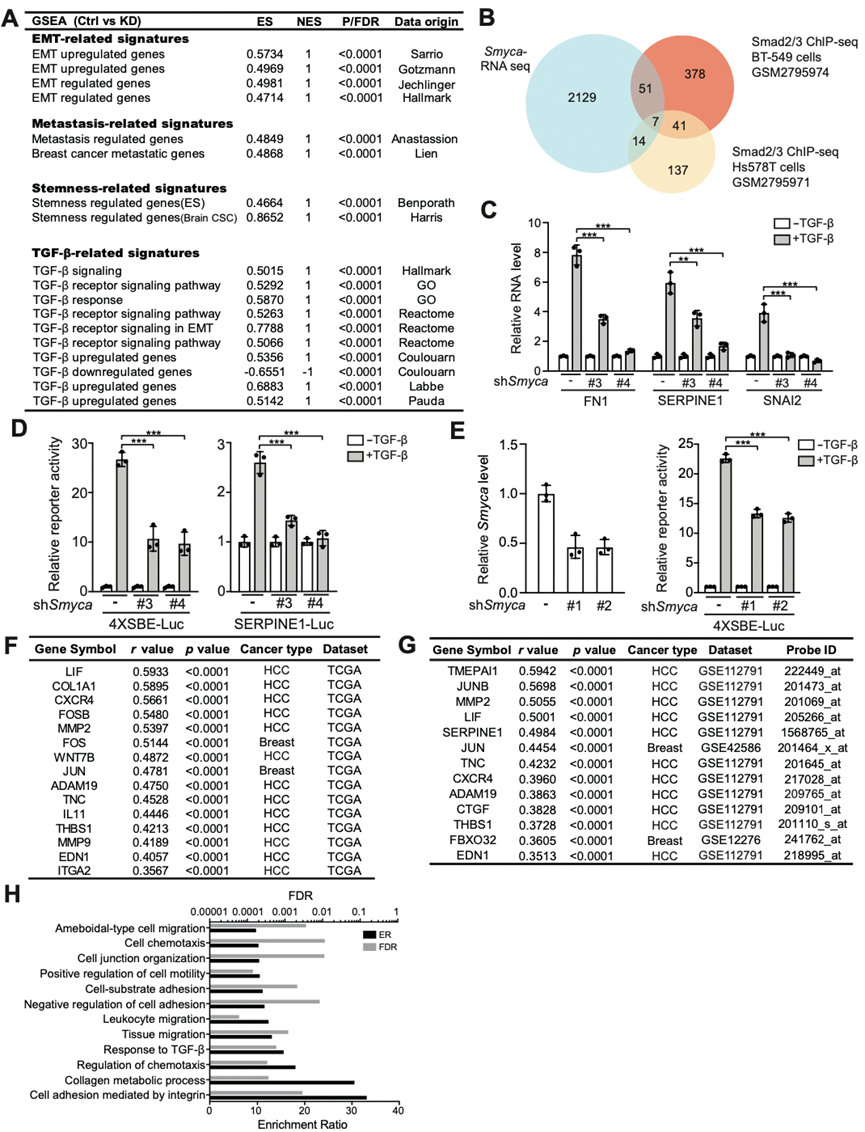


**Figure S3. Bioinformatics and cell-based analysis for the relation of *Smyca* to TGF-β signaling.** **(A)** Summary of the GSEA analysis for the match of *Smyca* signature with the indicated signatures. Data origin indicates the source database or the first author identifying the gene signature. (**B**) Intersection of *Smyca*-induced DEGs derived from MDA-MB-231 cells with Smad2/3 ChIP-seq data derived from Hs578T cells and BT-549 cells. The full list of overlapped genes is shown in Supplementary Table S5. **(C)** qRT-PCR analysis for the expression of indicated TGF-β target genes in Hs578T cells stably expressing *Smyca* shRNAs and treated with or without 5 ng/ml TGF-β for 48 hr. Data are normalized with that of untreated group in each cell. **(D, E)** Luciferase assay for the Smad-target reporters transfected into Hs578T cells **(D)** or Malaru cells **(E)** stably expressing *Smyca* shRNAs and treated with or without 5 ng/ml TGF-β for 24 hr. The *Smyca* knockdown efficiencies are shown on the left panel in **(E)**. Data in **(C)**, **(D)**, **(E)** are normalized with that of untreated control and presented as mean ± SD, n=3. *P* values are determined by one-way ANOVA with Tukey’s post hoc test, ***P*<0.01, ****P*<0.001. **(F, G)** Summary of the correlations of *Smyca* expression with the expression of indicated TGF-β target genes by analyzing HCC or breast cancer data sets from TCGA **(F)** or GEO **(G)** databases. Pearson’s coefficients and *P* values are indicated. **(H)** GO analysis using the set of TGF-β target genes with expression levels showing positive correlations with *Smyca* expression in HCC and/or breast cancers.

**Figure S4. *Smyca* binds MH1 domains of Smad3 and Smad4 without affecting their expression and Smad3 phosphorylation.** **(A)** Western blot analysis of Smad3 and Smad4 expression in MDA-MB-231 cells stably expressing *Smyca* shRNAs. **(B)** Western blot analysis for Smad3 phosphorylation in MDA-MB-231 cells stably expressing *Smyca* shRNAs and treated with or without 5 ng/ml TGF-β for 30 min. The blots are representative of three independent experiments and quantitative data are shown on the right. (**C**) Immunoprecipitation analysis of Smad3 and Smad4 interaction in MDA-MB-231 cells stably expressing indicated *Smyca* constructs and treated with or without 5 ng/ml TGF-β for 1 hr. **(D)** Luciferase assay for the Smad-responsive reporter in MDA-MB-231 cells transfected with indicated *Smyca* constructs and treated with or without 5 ng/ml TGF-β for 24 hr. The expression levels of *Smyca* are shown on the left panel. **(E)** Baculovirally purified Smad3, Smad4, and their MH1 deletion mutants bound on beads were incubated with biotinylated sense or antisense *Smyca*. The bound *Smyca* was analyzed by qRT-PCR. The equal inputs of recombinant proteins are shown on the right. Data in (**B**), **(D)** and **(E)** are mean ± SD, n=3. *P* values are determined by one-way ANOVA with Tukey’s post hoc test **(B, D)** or unpaired t-test **(E)**, ****P*<0.001; ns., not significant.

**Figure S5. *Smyca* is a Smad target and mediates a positive feedback control of TGF-β signaling.** **(A)** qRT-PCR analysis of *Smyca* expression in MDA-MB-231 cells stably expressing Smad3 or Smad4 shRNAs and treated with or without 5 ng/ml TGF-β for 24 hr. The knockdown efficiencies of Smad3 and Smad4 shRNAs are shown on the left and middle panels, respectively. **(B)** qRT-PCR analysis of TGF-β target gene expression in MDA-MB-231 cells stably expressing *Smyca* shRNAs and treated with or without 5 ng/ml TGF-β for indicated time points. Data are normalized with that of untreated group (0 h). Data in **(A)** and **(B)** are mean ± SD, n=3. *P* values are determined by unpaired t-test **(A)** or one-way ANOVA with Tukey’s post hoc test **(B)**, ****P*<0.001.

**Figure S6. *Smyca* promotes c-Myc transcription activity without affecting its expression or interaction with Max. (A)** qRT-PCR analysis of the expression of indicated c-Myc target genes in Malaru cells stably expressing control or *Smyca* shRNA. Data are normalized with that of control cells. **(B, C)** Luciferase assay for a c-Myc-reponsive reporter transfected into MDA-MB-231 cells stably expressing *Smyca* or mutant **(B)** or *Smyca* shRNAs **(C)**. The expression levels of *Smyca* and its mutant are shown on the left panel in **(B)**. **(D)** Summary of the correlations of *Smyca* expression with the expression of indicated c-Myc target genes by analyzing breast cancer and HCC data sets from TCGA. Pearson’s coefficients and *P* values are indicated. (**E**) Maping the *Smyca*-binding region in c-Myc. Top: Schematic presentaton of c-Myc domains. The various GFP-c-Myc truncated proteins were purified from transfected 293T cells and incubated with biotinylated sense or antisense *Smyca*. The bound *Smyca* was analyzed by qRT-PCR. Data are normalized with that from GFP only control. The input levels of various GFP fusion proteins are shown on the right and marked by arrows. **(F)** Western blot analysis of c-Myc expression in indicated cells stably expressing *Smyca* or *Smyca* shRNAs. **(G)** Immunoprecipitation analysis of c-Myc/Max complex formation in MDA-MB-231 cells stably expressing *Smyca* or *Smyca* shRNA. Data in **(A), (B), (C), (E)** are mean ± SD, n=3. *P* values are determined by unpaired t-test **(A)**, or one-way ANOVA with Tukey’s post hoc test **(B, C, E)**, **P*<0.05, ***P*<0.01, ****P*<0.001.

**Figure S7. *Smyca*-promoted c-Myc singaling neutralizes the growth inhibitory effect of *Smyca*-promoted TGF-β signaling.** (**A**) Immunoprecipitation analysis of c-Myc/Smad complex formation in MDA-MB-231 cells stably expressing *Smyca* and treated with 5 ng/ml TGF-β for 2 hr. **(B, C)** Luciferase assay for a Smad-responsive reporter (**B**) or c-Myc-responsive reporter (**C**) transfected into MDA-MB-231 cells together with indicated *Smyca* constructs and treated with or without 5 ng/ml TGF-β for 24 hr. The expression levels of *Smyca* (1-500) and (1001-1500) fragments are shown on the left and middle panels in (**B**), respectively. **(D, H)** Cell proliferation assay of MCF7 cells stably expressing *Smyca* and treated with 75 µM 10058-F4 for 24 hr **(D)** or NTU-BL cells stably expressing *Smyca* and treated with 5 µM SB431542 or 75 µM 10058-F4 for 24 hr **(H)**. **(E, F)** ChIP analysis for Smad3, Smad4, and c-Myc binding to the promoter regions of *CDKN2B* **(E)** and *CDKN1A* **(F)** genes in MDA-MB-231 cells stably expressing *Smyca* and treated with 5 µM SB431542 and/or 150 µM 10058-F4 for 2 hr. **(G, I)** qRT-PCR analysis of the expression of indicated genes in MCF7 **(G)** or NTU-BL **(I)** cells stably expressing *Smyca* and treated with 5 µM SB431542 or 75 µM 10058-F4 for 24 hr. *Smyca* expression levels are shown on the left panel of **(I)**. Data in (**B**), (**C**), (**D**), (**E**), (**F**), (**G**), (**H**), (**I**) are mean ± SD, n=3. *P* values are determined by one-way ANOVA with Tukey’s post hoc test, **P*<0.05, ***P*<0.01, ****P*<0.001.

**Figure S8. Downregulation of the expression of *Smyca*, Smad targets, and c-Myc targets by *Smyca* gapmer ASO**. **(A, B, D)** qRT-PCR analysis of *Smyca* or indicated mRNAs in MDA-MB-231 cells transfected with *Smyca* gapmer ASO **(A)**, LM6 cells treated with indicated doses of NPs carrying *Smyca* gapmer ASO or control gapmer **(B)**, or LM6 tumor-bearing mice treated with NPs carrying *Smyca* gapmer ASO or control NPs **(D)**. **(C)** The morphology, size and weight of primary tumors taken from the sacrifice day for experiment shown in **Fig. 7F**. Data in all panels are mean ± SD, n=3 **(A, B, D)** or 4 **(C)**. *P* values are determined by unpaired t-test, **P*<0.05, ***P*<0.01, ****P*<0.001.

**Table S1. Antibody details**

| **Protein** | **Vendor** | **Cat. number** | **Species** | **Titer** |
| --- | --- | --- | --- | --- |
| ZO-1 | GeneTex | GTX108613 | Rabbit | WB (1:1000) |
| Twist 1/2 | GeneTex | GTX127310 | Rabbit | WB (1:1000) |
| E-cadherin | Abcam | ab40772 | Rabbit | WB (1:1000) |
| SNAI1 | Cell Signaling | 3895 | Rabbit | WB (1:3000) |
| SNAI2 | Cell Signaling | 9585 | Rabbit | WB (1:1000) |
| Vimentin | Cell Signaling | 5741 | Rabbit | WB (1:500) |
| Vimentin | Sigma-Aldrich | V6389 | Mouse | WB (1:1000) |
| Smad2/3 | Cell Signaling | 3102 | Rabbit | WB (1:1000) |
| pSmad2 (S465/467)/pSmad3 (S423/425) | Cell Signaling | 8828 | Rabbit | WB (1:1000) |
| Smad3 | Cell Signaling | 9523 | Rabbit | IP (1:100) WB (1:1000) |
| Smad4 | Cell Signaling | 4653S | Rabbit | IP (1:100) WB (1:1000) |
| c-Myc | Cell Signaling | 9402 | Rabbit | IP (1:100) WB (1:1000) |
| c-Myc | Cell Signaling | 5605 | Rabbit | WB (1:1000) |
| Max | Cell Signaling | 4739 | Rabbit | WB (1:500) |
| Max | Santa Cruz | SC-8011 | Mouse | IP (1:150) |
| EasyBlot anti-Rabbit IgG (HRP) | GeneTex | 221666-01 | Sheep | WB (1:1000) |
| GAPDH | GeneTex | GTX100118 | Rabbit | WB (1:10000) |
| β−Actin | GeneTex | GTX110564 | Rabbit | WB (1:1000) |
| α−Tubulin | GeneTex | GTX112141 | Rabbit | WB (1:1000) |
| anti-Rabbit IgG H&L | Abcam | ab171870 | Rabbit | IP (control IgG) |
| Rabbit IgG HRP | GE Healthcare | NA934 | Donkey | WB (1:5000) |
| Mouse IgG HRP | GE Healthcare | NA931 | Sheep | WB (1:5000) |
| APC Mouse anti-human CD44 | BD bioscience | 559942 | Mouse | Flow (1:50) |
| APC Mouse IgG2b κ Isotype Control | BD bioscience | 555745 | Mouse | Flow (1:50) |
| PE Mouse anti-human CD24 | BD bioscience | 555428 | Mouse | Flow (1:50) |
| PE Mouse IgG2a κ Isotype Control | BD bioscience | 55574 | Mouse | Flow (1:50) |
| TRRAP | Invitrogen | PA5-78246 | Rabbit | WB (1:1000) |

**Table S2. siRNA, shRNA and Gapmer sequences**

| **shRNA/siRNA/Gapmer** | **Target sequence** | **Source (Identifiers)** |
| --- | --- | --- |
| **shRNA** |  |  |
| **sh*Luciferase*** | CTTACGCTGAGTACTTCGAGTG | National RNAi Core Facility, Academia Sinica, Taiwan |
| **sh*Control*** | CAACAAGATGAAGAGCACCAAA | National RNAi Core Facility, Academia Sinica, Taiwan |
| **sh*Smyca*#1** | GCATAGATAGGTGGGTGAGTG | BLOCK-iT™ RNAi Designer |
| **sh*Smyca*#2** | GCTGATGCTTGGAGCAGAGAT | BLOCK-iT™ RNAi Designer |
| **sh*Smyca*#3** | GGGCATGGAACAAGTTCCTTGTG | BLOCK-iT™ RNAi Designer |
| **sh*Smyca*#4** | GGCATGGAACAAGTTCCTTGT | BLOCK-iT™ RNAi Designer |
| **sh*Smad3*#1** | GCCTCAGTGACAGCGCTATTT | National RNAi Core Facility, Academia Sinica, Taiwan |
| **sh*Smad3*#2** | GAGCCTGGTCAAGAAACTCAA | National RNAi Core Facility, Academia Sinica, Taiwan |
| **sh*Smad4*#1** | GTACTTCATACCATGCCGATT | National RNAi Core Facility, Academia Sinica, Taiwan |
| **sh*Smad4*#2** | CAGATTGTCTTGCAACTTCAG | National RNAi Core Facility, Academia Sinica, Taiwan |
| **siRNA** |  |  |
| **si*Myc*** | CCTGAGACAGATCAGCAACAA | Dharmacon |
| **si*Control*** | TTCTCCGAACGTGTCACGTTT | Dharmacon |
| **Gapmer** |  |  |
| **Gapmer *Control*** | GCTCCCTTCAATCCAA | Qiagen |
| **Gapmer *Smyca*** | CGCATTGGGAGACTAA | Qiagen |

**Table S3. Sequences of PCR primers**

| **Gene name** |  | **Sequence 5' to 3'** |
| --- | --- | --- |
| ***miR-23a*** | RT/R | 5’-GTTGGCTCTGGTGCAGGGTCCGAGGTATTCG CACCAGAGCCAACGGAAAT-3’ |
| ***miR-24-2*** | RT/R | 5’-GTTGGCTCTGGTGCAGGGTCCGAGGTATTCG CACCAGAGCCAACCTGTGT-3’ |
| ***miR-24*** | RT/R | 5’-GTTGGCTCTGGTGCAGGGTCCGAGGTATTCG CACCAGAGCCAACCTGTTC-3’ |
| ***miR-27a*** | RT/R | 5’-GTTGGCTCTGGTGCAGGGTCCGAGGTATTCG CACCAGAGCCAACGCGGAA-3’ |
| ***miR-23a*** | F | 5’-GCGTCCATCACATTGCCAGGG-3’ |
| ***miR-24-2*** | F | 5’-CGTCGTGCCTACTGAGCTGAA-3’ |
| ***miR-24*** | F | 5’-TTGGGTGGCTCAGTTCAGCAG-3’ |
| ***miR-27a*** | F | 5’-CGTTCCGTTCACAGTGGCTAAG-3’ |
| ***miRNA* universal** | R | 5’-GTGCAGGGTCCGAGGT-3’ |
|  |  |  |
| ***GAPDH*** | F | 5’-TGTTGCCATCAATGACCCCTT-3’ |
|  | R | 5’-CTCCACGACGTACTCAGCG-3’ |
| ***18S rRNA*** | F | 5’-CAATTACAGGGCCTCGAAAG-3’ |
|  | R | 5’-AAACGGCTACCACATCCAAG-3’ |
| ***Smyca (*for full length*)*** | F | 5’-TTGAATTCCTGGGCACAAGT-3’ |
|  | R | 5’-CACACCTGAGCCACCTGTAA-3’ |
| ***Smyca* (for mutants)** | F | 5’-GGACTGAGGAGGCACACTGAA-3’ |
|  | R | 5’-ACTTGAACAACAGACATGACACACA-3’ |
| ***Neat1*** | F | 5’-TCGGGTATGCTGTTGTGAAA-3’ |
|  | R | 5’-TGACGTAACAGAATTAGTTCTTACCA-3’ |
| ***SERPINE1*** | F | 5’-GTTTCAGGCTGACTTCACGA-3’ |
|  | R | 5’-ATGACAGCTGTGGATGAGGA-3’ |
| ***SNAI2*** | F | 5’-CATGCCTGTCATACCACAAC-3’ |
|  | R | 5’-GGTGTCAGATGGAGGAGGG-3’ |
| ***Smad3*** | F | 5’-CATCGAGCCCCAGAGCAATA-3’ |
|  | R | 5’-GTGGTTCATCTGGTGGTCACT-3’ |
| ***Smad4*** | F | 5’-CCAATCATCCTGCTCCTGAGT-3’ |
|  | R | 5’-CCAGAAGGGTCCACGTATCC-3’ |
| ***MMP2*** | F | 5’-CACCCTGGAGCGAGGGTAC-3’ |
|  | R | 5’-CTGATTAGCTGTAGAGCTGAAGGC-3’ |
| ***MMP9*** | F | 5’-TGTACCGCTATGGTTACACTCG-3’ |
|  | R | 5’-GGCAGGGACAGTTGCTTCT-3’ |
| ***SMAD7*** | F | 5’-CCTTAGCCGACTCTGCGAACTA-3’ |
|  | R | 5’-TGCATAAACTCGTGGTCATTGG-3’ |
| ***FN1*** | F | 5’-CATCGAGCGGATCTGGCCC-3’ |
|  | R | 5’-GCAGCTGACTCCGTTGCCCA-3’ |
| ***C-JUN*** | F | 5’-GGAAACGACCTTCTATGACGATGCCC-3’ |
|  | R | 5’-GGCGCGCACGAAGCCCTCGGCGAACC-3’ |
| ***HK2*** | F | 5’-TGCCACCAGACTAAACTAGACG-3’ |
|  | R | 5’-CCCGTGCCCACAATGAGAC-3’ |
| ***LDHA*** | F | 5’-TTGACCTACGTGGCTTGGAAG-3’ |
|  | R | 5’-GGTAACGGAATCGGGCTGAAT-3’ |
| ***PDK1*** | F | 5’-ACTTCGGATCAGTGAATGCTTG-3’ |
|  | R | 5’-ACTCTTGCCGCAGAAACATAAA-3’ |
| ***GPI*** | F | 5’-AGGCTGCTGCCACATAAGGT-3’ |
|  | R | 5’-AGCGTCGTGAGAGGTCACTTG-3’ |
| ***LSS*** | F | 5’-ACATTGAGGATAAGTCCACCGT-3’ |
|  | R | 5’-TCGTACCAGGTCAGGATCGTC-3’ |
| ***SCD*** | F | 5’-GCAGGACGATATCTCTAGCT-3’ |
|  | R | 5’-GTCTCCAACTTATCTCCTCCATTC-3’ |
| ***SERBP1*** | F | 5’-TAGACCGATTATTGACCGACCT-3’ |
|  | R | 5’-GTTTGCCACGAGAATCAAATCC-3’ |
| ***FASN*** | F | 5’-TCGTGGGCTACAGCATGGT-3’ |
|  | R | 5’-GCCCTCTGAAGTCGAAGAAG-3’ |
| ***ACLY*** | F | 5’-CAGCAGGACAGCATCTTTTTC-3’ |
|  | R | 5’-TGGACTTGGGACTGAATCTTG-3’ |
| ***C1QBP*** | F | 5’-AGAAGCGAAATTAGTGCGGAA-3’ |
|  | R | 5’-CCACGAAATTGGGAGTTGATGTC-3’ |
| ***c-Myc*** | F | 5’-GCCACGTCTCCACACATCAG-3’ |
|  | R | 5’-TCTTGGCAGCAGGATAGTCCTT-3’ |
| ***CDKN2B*** | F | 5’-CACCCCCACCCACCTAATTC-3’ |
|  | R | 5’-TGAGTG TCGAGGGCCAGATA-3’ |
| ***CDKN1A*** | F | 5’-CCTCATCCCGTGTTCTCCTTT-3’ |
|  | R | 5’-GTACCACCCAGCGGACAAGT-3’ |
| ***CCNA1*** | F | 5’-TCCAAGAGGACCAGGAGAATATCA-3’ |
|  | R | 5’-TCCTCATGGTAGTCTGGTACTTCA-3’ |
| ***LINC00941*** | F | ACCACTACACTCAGCCAAATAC |
|  | R | GGCTATCAACTGTCTCCTTTAGAC |
| ***ELIT-1*** | F | GAGGCTGCAAGTTCAAGGTC |
|  | R | CACAGGGAGGATGTGTTGTG |
| ***Smad7* promoter** | F | 5’-TAGAAACCCGATCTGTTGTTTGCG-3’ |
|  | R | 5’-CCTCTGCTCGGCTGGTTCCACTGC-3’ |
| ***SERPINE1* promoter** | F | 5’-GCAGGACATCCGGGAGAGA-3’ |
|  | R | 5’-CCAATAGCCTTGGCCTGAGA-3’ |
| ***MMP2* promoter** | F | 5’-TCCCAGGCCTGCCCATGTCA-3’ |
|  | R | 5’-GGAGCTGGTGGGTGGAAAGCC-3’ |
| ***SNAI2* promoter** | F | 5’-CTGCACCACATCTGGAAGCCAG-3’ |
|  | R | 5’-CCAATCA CAGCTGAGAGGTTCAG-3’ |
| ***GAPDH* promoter** | F | 5’-AGCTCAGGCCTCAA GACCTT-3’ |
|  | R | 5’-AAGAAGATGCGGCTGACTGT-3’ |
| ***HK2* promoter** | F | 5’-CCGCAGGTAGTCAGGGATTG-3’ |
|  | R | 5’-GCAGCCACGATTCTCTCC-3’ |
| ***LDHA* promoter** | F | 5’-CACCCGGGCCTCTCCAGTGC-3’ |
|  | R | 5’-GGGCCTTAAGTGGAACAGCTATGCTGAC-3’ |
| ***FASN* promoter** | F | 5’-CGTGTGCGGGATGGGAATGCTTG-3’ |
|  | R | 5’-GGGTCCGTCCGTCCTTCCGC-3’ |
| ***PDK1* promoter** | F | 5’-ACGACCTCCCGCCTCCCGGC-3’ |
|  | R | 5’-TGCCCACGCTCGGCCTCCCA-3’ |
| ***GPI* promoter** | F | 5’-GCGGGCAGCCGCTCGCTCTG-3’ |
|  | R | 5’-CCTACCTGCGCGCACCGCCG-3’ |
| ***Smyca* promoter SBE#1** | F | 5’-ACCCACCACATCCCTCCTCCA-3’ |
|  | R | 5’-CCTTCCCTGGGTGGGCTGATG-3’ |
| ***Smyca* promoter SBE#2** | F | 5’-GCAGCAGGATGGCAGGCAGA-3’ |
|  | R | 5’-TTGTGAGCAGGGTCCACACCAAG-3’ |
| ***CDKN1A* promoter SBE** | F | 5’-CAAGGCTTCTGCAAATATGGACC-3’ |
|  | R | 5’-CTCAGCATCAGTGTTACCAAC-3’ |
| ***CDKN2B* promoter SBE** | F | 5’-TGGTGGCTCCCTTGTGACCGA-3’ |
|  | R | 5’-AGCCCAAGTACTGCCTGGGGA-3’ |
| ***CDKN1A* promoter MRE** | F | 5’-TGGGCGCGGATTCGCCGAGGCACC-3’ |
|  | R | 5’-GCGAACACGCATCCTCGCGGACACGCA-3’ |
| ***CDKN2B* promoter MRE** | F | 5’-ACCAGCGGGCGCGCCTGGATT-3’ |
|  | R | 5’-AGACGCCGGCCCCTTGGCCC-3’ |

F: Forward; R: Reverse; RT: Reverse transcription; SBE: Smad binding element; MRE: c-Myc responsive element

**Table S4. ChIRP probe sequences**

| **Probe name** | **Sequence (5' to 3')** |
| --- | --- |
| ***LacZ*** |  |
| **ChIRP *LacZ*‐1** | TAGCCAGCTTTCATCAACAT |
| **ChIRP *LacZ*‐2** | AGCAGCAGACCATTTTCAAT |
| **ChIRP *LacZ*‐3** | GTGTGGGCCATAATTCAATT |
| **ChIRP *LacZ*‐4** | CGGCAGCCGTTATTATTATT |
| **ChIRP *LacZ*‐5** | GAAACTGTTACCCGTAGGTA |
| **ChIRP *LacZ*‐6** | CACGGCGTTAAAGTTGTTCT |
| **ChIRP *LacZ*‐7** | GGATCGACAGATTTGATCCA |
| **ChIRP *LacZ*‐8** | GTAGTTCAGGCAGTTCAATC |
| **ChIRP *LacZ*‐9** | CAACGGTAATCGCCATTTGA |
| **ChIRP *LacZ*‐10** | TGCAAGGCGATTAAGTTGGG |
|  |  |
| ***Smyca*** |  |
| **ChIRP *Smyca*-1** | CTCTGCTCCAAGCATCAG |
| **ChIRP *Smyca*-2** | CCTGCAGCACACATTTGG |
| **ChIRP *Smyca*-3** | CTCACCCACCTATCTATG |
| **ChIRP *Smyca*-4** | CAGGCTTGGGAGAGGAGG |
| **ChIRP *Smyca*-5** | TTTAACGGTCTGGAGGCC |
| **ChIRP *Smyca*-6** | GTTAGAAAGCTGCAGGGC |
| **ChIRP *Smyca*-7** | CTGCTTGTGTCCCTTCTG |
| **ChIRP *Smyca*-8** | AGAGTCCTAACTCTTCCC |
| **ChIRP *Smyca*-9** | TCAGTCCCACCCAGAAAA |
| **ChIRP *Smyca*-10** | CTTCTGTCCACGACTACC |
| **ChIRP *Smyca*-11** | GAAGTCAGGGCAACTTTT |

**Table S5. List of genes that are regulated by *Smyca* and bound by Smad2/3**

| **Gene ID** | **Gene Symbol** | **Hs578T ChIP-seq** | **BT549 ChIP-seq** |
| --- | --- | --- | --- |
| ENSG00000134107 | BHLHE40 | YES | YES |
| ENSG00000114315 | HES1 | YES | YES |
| ENSG00000168264 | IRF2BP2 | YES | YES |
| ENSG00000134333 | LDHA | YES | YES |
| ENSG00000160209 | PDXK | YES | YES |
| ENSG00000137834 | SMAD6 | YES | YES |
| ENSG00000026025 | VIM | YES | YES |
| ENSG00000196526 | AFAP1 | YES | NO |
| ENSG00000167123 | CERCAM | YES | NO |
| ENSG00000168209 | DDIT4 | YES | NO |
| ENSG00000165323 | FAT3 | YES | NO |
| ENSG00000054598 | FOXC1 | YES | NO |
| ENSG00000185340 | GAS2L1 | YES | NO |
| ENSG00000115738 | ID2 | YES | NO |
| ENSG00000165055 | METTL2B | YES | NO |
| ENSG00000219481 | NBPF1 | YES | NO |
| ENSG00000175745 | NR2F1 | YES | NO |
| ENSG00000156858 | PRR14 | YES | NO |
| ENSG00000174136 | RGMB | YES | NO |
| ENSG00000108239 | TBC1D12 | YES | NO |
| ENSG00000101255 | TRIB3 | YES | NO |
| ENSG00000108846 | ABCC3 | NO | YES |
| ENSG00000139211 | AMIGO2 | NO | YES |
| ENSG00000165801 | ARHGEF40 | NO | YES |
| ENSG00000113273 | ARSB | NO | YES |
| ENSG00000105974 | CAV1 | NO | YES |
| ENSG00000122863 | CHST3 | NO | YES |
| ENSG00000165376 | CLDN2 | NO | YES |
| ENSG00000135924 | DNAJB2 | NO | YES |
| ENSG00000125037 | EMC3 | NO | YES |
| ENSG00000134824 | FADS2 | NO | YES |
| ENSG00000269190 | FBXO17 | NO | YES |
| ENSG00000089327 | FXYD5 | NO | YES |
| ENSG00000013588 | GPRC5A | NO | YES |
| ENSG00000138641 | HERC3 | NO | YES |
| ENSG00000049860 | HEXB | NO | YES |
| ENSG00000137309 | HMGA1 | NO | YES |
| ENSG00000196639 | HRH1 | NO | YES |
| ENSG00000240875 | LINC00886 | NO | YES |
| ENSG00000251562 | MALAT1 | NO | YES |
| ENSG00000069020 | MAST4 | NO | YES |
| ENSG00000082126 | MPP4 | NO | YES |
| ENSG00000066697 | MSANTD3 | NO | YES |
| ENSG00000125148 | MT2A | NO | YES |
| ENSG00000070614 | NDST1 | NO | YES |
| ENSG00000119408 | NEK6 | NO | YES |
| ENSG00000100906 | NFKBIA | NO | YES |
| ENSG00000166741 | NNMT | NO | YES |
| ENSG00000182446 | NPLOC4 | NO | YES |
| ENSG00000157168 | NRG1 | NO | YES |
| ENSG00000138496 | PARP9 | NO | YES |
| ENSG00000196155 | PLEKHG4 | NO | YES |
| ENSG00000173457 | PPP1R14B | NO | YES |
| ENSG00000186350 | RXRA | NO | YES |
| ENSG00000197747 | S100A10 | NO | YES |
| ENSG00000196754 | S100A2 | NO | YES |
| ENSG00000130066 | SAT1 | NO | YES |
| ENSG00000099194 | SCD | NO | YES |
| ENSG00000157933 | SKI | NO | YES |
| ENSG00000162241 | SLC25A45 | NO | YES |
| ENSG00000104635 | SLC39A14 | NO | YES |
| ENSG00000171992 | SYNPO | NO | YES |
| ENSG00000158710 | TAGLN2 | NO | YES |
| ENSG00000137801 | THBS1 | NO | YES |
| ENSG00000253304 | TMEM200B | NO | YES |
| ENSG00000196428 | TSC22D2 | NO | YES |
| ENSG00000117472 | TSPAN1 | NO | YES |
| ENSG00000119048 | UBE2B | NO | YES |
| ENSG00000186591 | UBE2H | NO | YES |
| ENSG00000169764 | UGP2 | NO | YES |
| ENSG00000188064 | WNT7B | NO | YES |
| ENSG00000101966 | XIAP | NO | YES |
